# Inhibition of lactate-histone lactylation mediated cardiomyocyte-fibroblast communication mitigates cardiac fibrosis in cardiomyopathy models with Hippo pathway activation

**DOI:** 10.1038/s41419-026-09137-7

**Published:** 2026-08-01

**Authors:** Li-Ye Jia, Xia-Xia Hai, Zhi-Qiang Wu, Jing-Jing Li, Yi-Yi Yang, Meng-Chen Hou, Lin-Hong Wu, Meng-Zhuan Han, Yi Zhang, Yungang Xu, Jie Deng, Bao-Chang Lai, Junichi Sadoshima, Xiu-Ling Deng, Zheng-Da Pang, Xiao-Jun Du, Gang She

**Affiliations:** 1 School of Nursing and Rehabilitation, Xi’an Medical University, Xi’an, China; 2 Department of Physiology and Pathophysiology, School of Basic Medical Sciences, Xi’an Jiaotong University Health Science Center, Xi’an, China; 3 Department of Cell Biology and Genetics, School of Basic Medical Sciences, Xi’an Jiaotong University Health Science Center, Xi’an, China; 4 Department of Pathology, Xi’an Guangren Hospital, Affiliated to Xi’an Jiaotong University Health Science Center, Xi’an, China; 5 Department of Cardiology, The Second Affiliated Hospital of Xi’an Jiaotong University, Xi’an, China; 6 Cardiovascular Research Centre, School of Basic Medical Sciences, Xi’an Jiaotong University Health Science Center, Xi’an, China; 7 Department of Cell Biology and Molecular Medicine, Rutgers New Jersey Medical School, Newark, USA; 8 Baker Heart and Diabetes Institute, Melbourne, Australia

**Keywords:** Cardiomyopathies, Experimental models of disease

## Abstract

Metabolic remodeling is a fundamental mechanism that underlies cardiomyopathy and heart failure. Preclinical and clinical studies in diverse types of heart disease have provided evidence for the activation of the myocardial Hippo pathway and that cardiomyocyte-specific Hippo pathway activation or YAP-TEAD1 inactivation drives cardiomyocyte mitochondrial damage, metabolic abnormalities and fibrosis. Here we studied whether intervention of myocardial lactate metabolism have therapeutic effect on cardiac fibrosis in cardiomyopathy models. In a Hippo pathway-activated mouse model (Mst1-cTG), single-nucleus RNA-sequencing (snRNA-seq) revealed activated fibroblasts while cardiomyocytes displayed downregulation of gene sets of mitochondrial metabolism and augmented HIF-1 signaling. Seahorse assay in isolated cardiomyocytes from adult TG mice showed impaired oxidative respiratory capacity together with enhanced glycolysis and lactate production. In TG mouse myocardium, fibroblast histone H3 lysine 18 lactylation (H3K18la), regulated by p300, was elevated starting from young age. By CUT&Tag, H3K18la was found to be enriched in promoter regions of numerous fibrotic genes with enhanced transcription. Treatment of TG mice with inhibitors to suppress either glycolysis (PX-478 as HIF-1α inhibitor) or lactate production (FX-11 as LDHA inhibitor) reduced both myocardial lactate content and H3K18la level, and ameliorated cardiac fibrosis and dysfunction. Similar efficacy was achieved in vitro or in vivo by fibroblast p300 knockdown using siRNA or AAV9. Treatment with FX-11 was similarly effective in inhibiting histone lactylation and fibrosis in mice subjected to chronic ischemia-reperfusion. Finally, we integrated our snRNA-seq and CUT&Tag studies to identify RUNX2 as a crucial transcription factor that promotes fibrotic gene expression in concert with H3K18la levels. Treatment with RUNX2 inhibitor CADD522 in TG mice improved heart dysfunction and fibrosis. In the setting of myocardial Hippo pathway activation, inhibiting the lactate-histone lactylation-RUNX2 axis effectively attenuated cardiac fibrosis.

**Figure:**
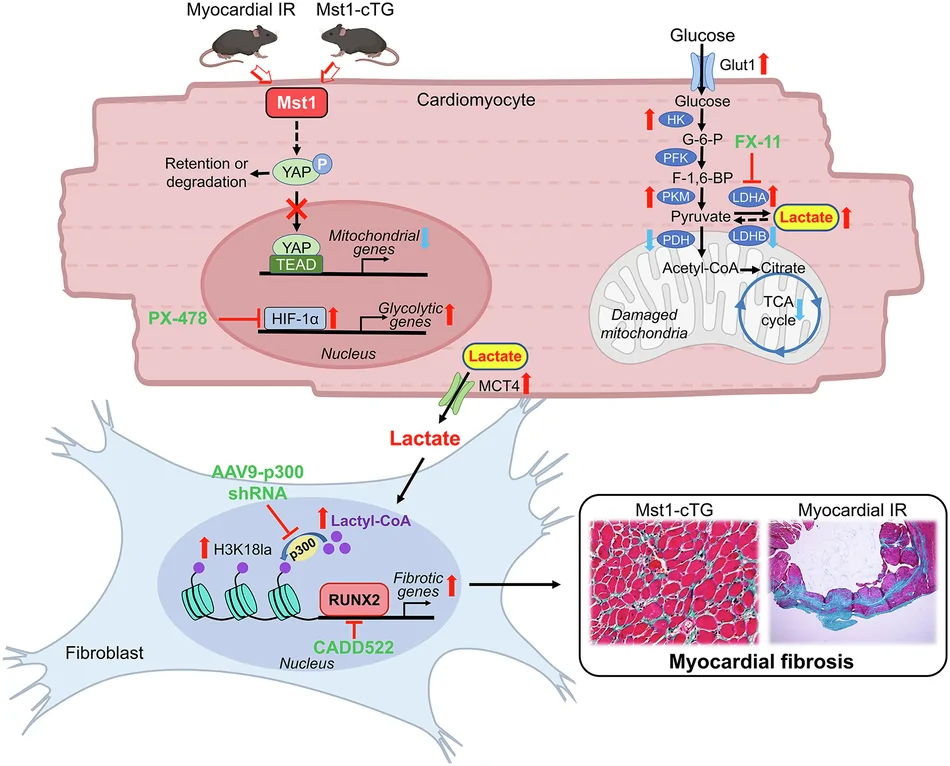
Myocardial Hippo pathway activation promotes the production and release of lactate that acts in a paracrine signaling fashion to activate fibroblasts through upregulation of fibroblast H3K18la and enhancing the enrichment of RUNX2 to the promoter regions of fibrotic genes with enhanced gene transcription, ultimately leading to cardiac fibrosis. Suppressing the lactate-histone lactylation-RUNX2 axis is protective against cardiac fibrosis.

Myocardial Hippo pathway activation promotes the production and release of lactate that acts in a paracrine signaling fashion to activate fibroblasts through upregulation of fibroblast H3K18la and enhancing the enrichment of RUNX2 to the promoter regions of fibrotic genes with enhanced gene transcription, ultimately leading to cardiac fibrosis. Suppressing the lactate-histone lactylation-RUNX2 axis is protective against cardiac fibrosis.

## Introduction

Cardiac fibrosis is a common pathophysiological process critical to the progression of heart disease. Fibrosis is characterized by excessive production and accumulation of extracellular matrix (ECM) in the myocardium following fibroblast activation [1]. Fibrosis increases myocardial stiffness to impede heart contraction and relaxation and also impairs cardiac electrophysiological functions, ultimately resulting in heart failure (HF) and arrhythmias [2]. The onset and progression of cardiac fibrosis involve multiple molecular signaling pathways and complex interactions among diverse cell types (such as fibroblasts, cardiomyocytes, inflammatory cells, and endothelial cells) [3], yet the precise mechanisms remain partially understood.

Metabolic flexibility of the heart is important in adapting to various physiological and pathological conditions that are associated with altered ATP demand [4]. The failing heart exhibits metabolic remodeling manifested by mitochondrial dysfunction with retarded utilization of fatty acids, amino acids and ketone bodies, and increased glycolysis [5]. However, the underlying mechanisms for metabolic remodeling remain poorly defined. Recent studies by this and others have demonstrated that Hippo pathway signaling is crucial for the regulation of myocardial mitochondrial structure and function [6–8]. The core molecular components of Hippo pathway signaling include upstream kinases mammalian sterile-20 like kinase1 (Mst1) and large tumor suppressor (Lats), and downstream transcription regulator yes-associated protein (YAP) and the transcription factor TEA domain family member1 (TEAD1). Canonical Mst1/Lats phosphorylation mediates YAP phosphorylation at Ser127 site to inhibit YAP nuclear translocation and its transcriptional regulatory activity with TEAD1, together with increased YAP cytoplasmic retention and subsequent degradation [9]. Accordingly, cardiomyocyte-specific gene targeting through either Hippo pathway activation (i.e., transgenic overexpression of Mst1, Mst1-cTG) or inhibition of YAP or TEAD1 (YAP-cKO, TEAD1-cKO or use of verteporfin to inhibit YAP-TEAD1) in mice all lead to dilated cardiomyopathy (DCM) manifested as chamber enlargement, dysfunction and significant fibrosis [6, 7, 10, 11]. Interestingly, in those models, the onset of DCM from the initiation of specific gene targeting is rapid, varying from 8 days (TEAD1-cKO) to 2–3 weeks (Mst1-cTG) or 8 weeks (YAP-cKO) [6, 11, 12]. Mechanistically, we showed in Mst1-cTG mice that cardiomyocytes exhibit significant mitochondrial damage, downregulation of mitochondrial genes and metabolic abnormalities, in particular impaired oxidative phosphorylation (OXPHOS), fatty acid β-oxidation (FAO) and branched-chain amino acid (BCAA) catabolism as well as enhanced glycolysis [6, 8]. Despite these observations, the precise mechanisms for enhanced pro-fibrotic signaling and fibrosis that are documented in cardiomyocyte-restricted Hippo pathway activation remain unclear.

Guided by our insights from studies with Mst1-cTG mice that showed elevated tissue levels of metabolites including lactate, BCAAs, ceramides and α-ketoglutarate [6, 13, 14], we hypothesize that cardiomyocyte metabolic disorder drives fibrosis. Of interest to our investigation is the role of lactate, a metabolite of cellular glycolysis, that has recently been implicated as a signaling molecule regulating cell function and phenotypic transition [15, 16]. This is achieved through protein lactylation, a process in which a lactate-derived lactyl moiety is covalently attached to lysine residues on target proteins via the action of specific Coenzyme A (CoA) transferases [17]. Recent studies have demonstrated that protein lactylation plays important roles in heart disease, including regulating repair and endothelial-mesenchymal transition (EndoMT) post-myocardial infarction (MI) [18, 19] and maintaining the structure and function of myofibrils in cardiomyocytes of a failing heart [20].

What remains unclear is whether cardiomyocyte-derived lactate might induce fibroblast activation and fibrosis through protein lactylation. Our previous study using Mst1-cTG mice showed that myocardial glycolysis was enhanced and lactate levels in both the myocardium and plasma were significantly increased, and this implied that lactate might be released from cardiomyocytes [6]. Here, we specifically explore the possibility that cardiomyocyte-derived lactate activates fibroblasts to promote cardiac fibrosis by inducing histone lactylation and the effect of lactate metabolism inhibition on fibrosis. We explored this issue through studies using Mst1-cTG mice, where we performed single-nucleus RNA sequencing (snRNA-seq) and H3K18la CUT&Tag assays. Moreover, we conducted a series of in vitro experiments using isolated cardiomyocytes and cultured cardiac fibroblasts prepared from adult mice. Furthermore, in vivo experiments were conducted to test targeted interventions on lactate production and lactylation in Mst1-cTG mice, as well as with mice subjected to chronic ischemia-reperfusion (IR).

## Materials and methods

### Animals, surgery and drug treatment

Male and female Mst1-cTG and non-TG littermate (nTG) mice bred on a C57BL/6J genetic background were used. There is no significant sex dimorphism in the DCM phenotype of Mst1-cTG mice (Supplementary Table S4). Overexpression of Mst1 transgene is governed by an α-myosin heavy chain (α-MHC) promoter, as previously reported [21, 22]. The genotypes of mice were determined by PCR of DNA samples from tail biopsies. In addition, thoracotomy was performed on wild-type male adult C57BL/6J mice to induce IR as the alternative model of myocardial fibrosis. As previously reported [23], the left coronary artery was occluded for 1 h then released for reperfusion. After surgery, the mice received subcutaneous injections of atipamezole (1 mg/kg) and carprofen analgesia (5 mg/kg) once daily for 48 h, and saline (0.5 mL). Operated mice recovered in a heat-supplemented environment before being moved to standard housing.

Three drugs tested *in viv*o included the LDHA inhibitor FX-11 (2 mg/kg dissolved in corn oil, i.p., once daily for 4 weeks) [24], HIF-1α inhibitor PX-478 (5 mg/kg dissolved in saline, i.p., once every second day for 4 weeks) [25], and RUNX2 inhibitor CADD522 (20 mg/kg dissolved in corn oil, i.p., twice a week for 4 weeks) [26]. At the end, animals were under deep anesthesia (isoflurane inhalation at 4%), and a blood sample was collected by cardiac puncture. The heart, lungs, liver, kidney, and spleen were harvested and weighed, and the left ventricle (LV) was isolated for assays. Body weight and the tibia length were measured. For studies involving comparison between experimental groups, animals were randomly allocated to each group. The sample size for each experimental group was chosen based on common practice in the field and our preliminary data to ensure reproducible observation of the phenotype. All experimental protocols were approved by the Biomedical Animal Ethics Committee of Health Science Center, Xi’an Jiaotong University, and conformed to the Guide for the Care and Use of Laboratory Animals published by the National Institutes of Health (No. 2022-624).

### Echocardiography

Echocardiography was performed using a Vevo 3100 ultrasound machine (Visualsonics Inc, Toronto, Canada) with a MX550D transducer, as described previously [27]. Mice were anesthetized under 1–2% isoflurane. Two-dimensional (2-D) short-axis image of the LV was obtained at the level of papillary muscles, and 2-D image-guided M-mode traces crossing the anterior and posterior walls were recorded. From the M-mode tracings, we measured LV wall thickness at diastole and systole (WTd, WTs), and the LV internal dimensions at diastole and systole (LVIDd, LVIDs). Fractional shortening (FS) was calculated as [(LVIDd-LVIDs)/LVIDd]×100%, and ejection fraction (EF) was calculated from LV diastolic and systolic volumes derived from LV dimensions. Wall thickening index (WTI) was calculated as [(WTs-WTd)/WTd]×100%, as previously described [28]. Images from 5 cardiac cycles were analyzed in a blinded fashion, and the average was used.

### Cleavage under targets and tagmentation (CUT&Tag) assay

As described previously [18], mouse LV tissues (5 mg) were prepared and washed in 100 µL washing buffer. Concanavalin A coated magnetic beads were prepared as described in the kit manual. Next, 10 µL of a suspension of activated beads was added to each sample and incubated at room temperature for 10 min. According to the instructions of the manufacturer, samples were then sequentially incubated with ConA Beads, primary antibody (anti-H3K18la antibody), secondary antibody (Goat anti-Rabbit IgG H&L) and Hyperactive PG-TN5/PA-TN5 Transposon and then fragmented. Fragmented DNA was extracted from the samples and amplified by PCR. CUT&Tag libraries were constructed using HyperactiveTM In-Situ ChIP Library Prep Kit (TD901, Vazyme, China) and sequenced on an Illumina NovaSeq platform, and 150-bp paired-end reads were generated. Fastp v 0.20.0 was used to remove adapters and low-quality reads. Align paired-end reads using Bowtie2 version 2.3.4.3. Duplicated reads were removed using Picard v2.18.17. Peak calling uses SEACR v1.3 with threshold of 0.01. Scatterplots, correlation plots, and heatmaps were displayed using deepTools v2.27.1. Annotation of peaks was performed using an R package ChIPseeker v1.12.1. MEME-ChIP v 5.0.5 was used to search for the binding site. Peaks with *M*-value > 0.2 and *P* value < 0.05 are defined as specific peaks.

### Cleavage under targets & release using nuclease (CUT&RUN) assay

The CUT&RUN Assay Kit for qPCR (CST, #86652) was used. Cultured adult mouse cardiac fibroblasts (1 × 10^5^) were prepared, washed in 100 µL washing buffer, and incubated with ConA beads for 10 min (25 °C). Samples were then incubated overnight (4 °C) with RUNX2 antibody. pG-MNase enzyme was added, and the samples were incubated for 1 h (4 °C), followed by washing twice with DIG washing buffer. Then, the samples were incubated with CaCl_2_ (10 mM) for 1 h (4 °C). After addition of 100 µL stop buffer, the samples were resuspended and incubated for 30 min (37 °C), plated on a magnet and liquid was drained. After gentle washes with 80% ethanol, DNA was eluted from the beads using double-distilled water. RT-qPCR tests were conducted in accordance with the manufacturer’s recommendations.

### Adeno-associated virus serotype 9 (AAV9) vector construction and administration

AAV9 was used for in vivo knockdown of the *p300* gene under the control of the periostin (postn) promoter to achieve fibroblast-restricted gene targeting. AAV9-postn-p300 shRNA and a negative control AAV9-postn-CTL shRNA were both constructed by OBiO Technology Company (Shanghai, China). Mice were injected through the tail vein with AAV9-postn-p300 shRNA or AAV9-postn-CTL shRNA at a dose of 1 × 10^12^ vg (100 µl)/animal. Six weeks after the injection, heart samples were collected for assays.

### Adult cardiomyocyte isolation and bioenergetic profiling with an Agilent Seahorse XF96 Analyzer

Adult mouse cardiomyocytes were isolated using a Langendorff perfusion method as described previously [8]. In brief, dissected hearts were perfused through an aortic cannula with calcium-free buffer followed by enzyme buffer solution. The ventricles were then minced in enzyme buffer. After gentle pipetting, samples were centrifuged at 500 r.p.m. for 1 min and cell pellets containing cardiomyocyte fractions were resuspended in stop buffer. Calcium content of the cell suspension was then stepwise adjusted to 1 mM.

The O_2_ consumption rate (OCR, pmol/min) and extracellular acidification rate (ECAR, mpH/min) in culture medium were both assessed in cells in real-time using a Seahorse XF96 Extracellular Flux Analyzer (Agilent Technologies) according to the manufacturer’s instructions. Cardiomyocytes were plated in laminin pre-coated 96-well Seahorse assay plates at a density of 1 × 10^5^ cells/well and 5000 cells/well, respectively. One hour before analysis, the medium was replaced with XF assay medium, and the cells were incubated for 1 h at 37 °C without CO_2_. For OCR measurements, after baseline measurements, 2 μM oligomycin (OM), 2 μM FCCP, and 1 μM rotenone and 1 μM antimycin A (Rot/AA) were sequentially injected into each well. The ECAR and OCR were normalized to the protein amount. For ECAR measurements, after baseline measurements, 10 mM glucose, 1 μM OM, and 50 mM 2-deoxy D-glucose (2-DG) were sequentially injected into each well.

### Isolation of adult mouse cardiac fibroblasts and RNA interference studies

Ventricular fibroblasts were isolated from adult male C57BL/6J mice using a standard collagenase digestion protocol as previously described [27]. Cells were cultured in DMEM medium supplemented with 15% FBS and a cocktail of antibiotics. Fibroblasts grown to approximately 70% confluence were transfected with p300-siRNA or RUNX2-siRNA and its control-siRNA, as described previously [29]. Specific siRNA molecules targeting p300 and RUNX2 were synthesized by Tsingke Biotechnology Co., Ltd (Beijing, China). The siRNA sequences of mouse p300 are: sense: 5′- CAGCCCAGUGAAUCAACCUTT-3′, antisense: 5′-AGGUUGAUUCACUGGGCUGTT-3′. The siRNA sequences of the mouse RUNX2 are: sense: 5′- GCACGCTATTAAATCCAAATT-3′, antisense: 5′- AATTTGGATTTAATAGCGTGC-3′. After 6-h transfection, cells were treated with or without 5 mM lactate for 48 h. After incubation, cells were harvested for analysis.

### Western blotting analysis

Total protein lysates were prepared from LV tissues, isolated adult cardiomyocytes or cultured cardiac fibroblasts using RIPA lysis buffer. A histone extraction kit (Saintbio, Shanghai, China) was used to prepare histone protein fractions from mouse LV tissues or cells. Protein samples (20 µg) were separated on a sodium dodecyl sulfate-polyacrylamide gel and transferred to a polyvinylidene fluoride membrane, as described previously [29]. The membranes were initially incubated with primary antibodies (see Table S1) overnight (4 °C), and then with horseradish peroxidase-conjugated secondary antibodies (1:10,000) for 1 h at room temperature. The bound antibodies were detected with an enhanced chemiluminescence detection system (Bio-Rad, USA) and quantified by densitometry using the ImageJ software. To ensure equal sample loading, the ratio of band intensity to α-tubulin, GAPDH, HSP90 or histone H3 was obtained to quantify the relative protein abundance of immunoblotted proteins of interest.

### RNA isolation and real-time quantitative PCR analysis

Total RNA was extracted from mouse LV tissues using TRIzol Reagent and RNA concentrations were measured spectrophotometrically. Each RNA sample was reverse-transcribed into cDNA with the RT kit according to the manufacturer’s protocol. Thermocycler conditions were as follows: 37 °C for 15 min, 85 °C for 5 s and 4 °C holding. RT-PCR amplification and detection were performed using SYBR Green dye following the manufacturer’s protocol. All samples were run in triplicate with an MJ MiniTM thermal cycler (Bio-Rad Laboratories Co., Ltd, Shanghai, China). Cycling conditions were as follows: 95 °C for 30 s followed by 39 cycles of amplification (95 °C denaturation for 5 s, 60 °C annealing for 30 s, 95 °C extension for 10 s), followed by the addition of melt curve conditions to ensure primer specificity. Fold changes in mRNA expression levels were measured using the comparative CT method (2^−ΔΔCt^). The primers used were products from Sangon Biotech Co., Shanghai, China and listed in Table S2.

### Chromatin accessibility assay

A chromatin accessibility kit was purchased from Abcam (ab185901), and the assay was performed according to the manufacturer’s instructions. Briefly, chromatin was isolated from cultured primary adult mouse heart fibroblasts (2 × 10^6^) and digested with nuclease mix. DNA samples were purified and analyzed by RT-qPCR. Primer sequences used for analyzing fibrotic gene promoter are listed in Supplementary Table S3.

### Immunofluorescent dual staining

Immunofluorescence dual-staining of LV sections or cultured fibroblasts was performed, as previously described [27]. In brief, formaldehyde fixed sections (5 μm) or cells were incubated overnight at 4 °C with combination of rabbit anti-H3K18la antibody (1:200) and mouse anti-DDR2 antibody (1:200) or combination of rabbit anti-RUNX2 antibody (1:200) and mouse anti-α-SMA antibody (1:200). After incubation with appropriate fluorescence-labeled secondary antibodies (1 h, room temperature), the sections or cells were finally incubated with DAPI for 10 min. Images were acquired with an Olympus FV3000 confocal microscope.

### Single-nucleus RNA sequencing and bioinformatics

The whole heart samples were harvested from 3-month-old male Mst1-cTG and nTG controls (*n* = 3 each). Heart nucleus extraction and snRNA-seq experiments were performed by the Novogene Co., Ltd, Tianjin, China (https://cn.novogene.com/) as previously described [8, 30].

Composition analysis [8]: To identify group differences in cell types, we applied the Bayesian method from scCODA v0.1.2. For this analysis, vascular smooth muscle cells were selected as a reference cell type with relatively invariant across samples.

Pathway analysis [8]: Statistically significant DE genes were used to perform pathway analysis via EnrichR (https://maayanlab.cloud/Enrichr/). Pathway enrichment values were downloaded as. csv files and plots were generated in GraphPad Prism.

Pseudotime trajectory analysis [18]: To predict the developmental trajectory of fibroblasts, cells were ordered in pseudotime using the Monocle (v2) package in R. The fibroblast gene expression matrix, extracted using the Subset function, was used as the input. Then, normalization was performed by estimateSizeFactors and estimateDispersions functions, and the reduceDimension command was used for dimensionality reduction with the method of “DDRTree”. The trajectories were visualized as 2D uniform manifold approximation and projection (UMAP) plots using the plot_cell_trajectory command with default parameters. The dynamic change of gene expression patterns in pseudotime was explored by using pseudotime dot plots.

Transcriptional factor (TF) activity analysis [31]: TF analysis was performed on the SCENIC R package. In brief, the gene expression matrix was used as the input, and the pipeline was implemented in three steps. First, the gene co-expression network was identified via GENIE3. Second, each module was pruned based on a regulatory motif near a transcription start site via RcisTarget. Third, the activity of each regulon was scored per each single cell via the Area Under Curve (AUC) scores using AUCell R package.

### Statistical analysis

The experiments have been replicated at least 3 times in the laboratory. No samples have been excluded. All data were presented as mean ± SEM. Results were analyzed using GraphPad Prism 9 software. The normality of data distribution was assumed based on the extensive use of these parametric tests in analogous experimental settings within the field. Student’s *t* test was used to compare differences between only two groups. Statistical comparisons among more than two groups were performed by one-way analysis of variance (ANOVA) with Bonferroni’s *post-hoc* test. For comparisons involving multiple factors, two-way ANOVA followed by Tukey’s multiple comparisons test was performed. All statistical tests were two-sided, and adjustments for multiple comparisons were applied where appropriate. A *p*-value of *P* < 0.05 was considered significantly different.

## Results

### SnRNA-seq revealed the coexistence of fibroblast activation and cardiomyocyte metabolic disorder in Mst1-cTG hearts

**Fig. 1:**
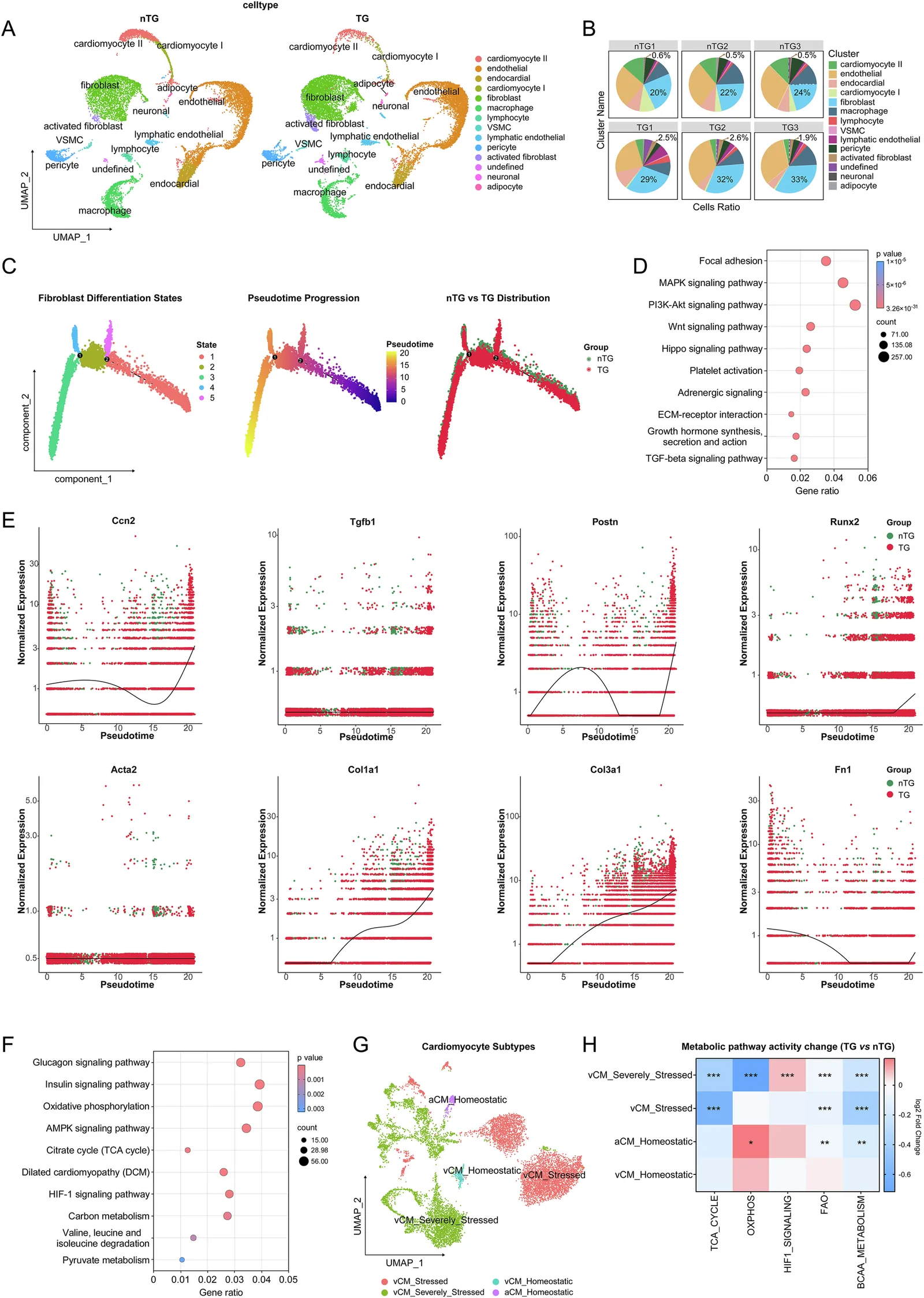
Single-nucleus RNA-sequencing analysis of adult Mst1-TG mouse heart. **A** Representative UMAP plot showing cell cluster difference among nTG and TG mouse heart samples (*n* = 3 each). **B** Pie plot showing Cell-type composition of individual samples with colors representing different cell types. Fibroblast ratio in each sample was marked. **C** Temporal analysis of fibroblast in nTG and TG mouse heart samples. **D** KEGG enrichment analysis for upregulated gene sets in Mst1-TG mouse fibroblast relative to nTG at the terminal stage of differentiation over pseudotime. **E** Dot plots depict fibrosis related gene expression over pseudotime. **F** KEGG enrichment analysis for changed gene sets in Mst1-TG mouse cardiomyocytes relative to nTG. **G** Representative UMAP plot showing cardiomyocyte subclusters in TG mouse heart which were separated by dilated cardiomyopathy and oxidative stress markers. **H** Heat map showing metabolic pathway score difference among TG mouse cardiomyocyte subclusters which was evaluated by GSEA analysis.

We performed snRNA-seq on whole heart samples from adult nTG and Mst1-cTG mice. After demultiplexing and mapping to a reference genome, 30 cell clusters were defined as 13 major cell types according to specific cell gene markers (Fig. S1A, B). UMAP representation of isolated nuclei revealed fibroblasts as the largest cell population secondary only to endothelial cells in the mouse heart, and that the density of fibroblasts and activated fibroblasts was higher in LVs of TG relative to nTG mice (Fig. 1A). Composition analysis indicated that the cell ratio of fibroblasts and activated fibroblasts expressed as an average was increased by 41% and 3.1-fold, respectively, in Mst1-cTG compared with nTG hearts (fibroblast: average 31.8% *vs*. 22.6%; activated fibroblast: average 2.33% *vs*. 0.56%, Fig. 1B). Pseudotime trajectory analysis of the developmental course of fibroblasts in Mst1-cTG relative to nTG hearts showed that fibroblasts were activated, and proliferated gradually over time leading to a greater amount of differentiated myofibroblasts in TG than nTG hearts at the end of differentiation trajectory (Fig. 1C). KEGG enrichment analysis found that signaling pathways related to focal adhesion, growth and ECM interaction were activated in end-stage differentiated fibroblasts of TG versus nTG hearts (Fig. 1D). Expression levels for fibrotic genes such as *Ccn2*, *Fn1*, *Col1a1*, *Col3a1* and *Runx2* in fibroblasts were higher with time in the TG group (Fig. 1E). These results indicate that fibroblast activation was enhanced under cardiomyocyte-specific Hippo pathway activation.

We performed KEGG enrichment and the data showed significant changes to pathways for glucose metabolism, OXPHOS, DCM and BCAA degradation in TG relative to nTG mouse cardiomyocytes (Fig. 1F). Next, we explored cell subtypes arising from the separation of atrial cardiomyocytes (aCMs) and ventricular cardiomyocytes (vCMs), with vCMs further divided into three stressed subtypes using DCM and oxidative stress markers (Figs. S1A, B and 1G). Through scoring the changes in the metabolic pathway in subtype cardiomyocytes, we found in vCMs of TG mice that the TCA cycle, OXPHOS, FAO and BCAA catabolism progressively declined whilst HIF-1α signaling concomitantly increased with the degree of stress (Fig. 1H). These data suggested that, in this model with myocardial Hippo pathway activation, metabolic remodeling of cardiomyocytes was accompanied by fibroblast activation.

### Augmented glycolysis and lactate production in Mst1-cTG cardiomyocytes

**Fig. 2:**
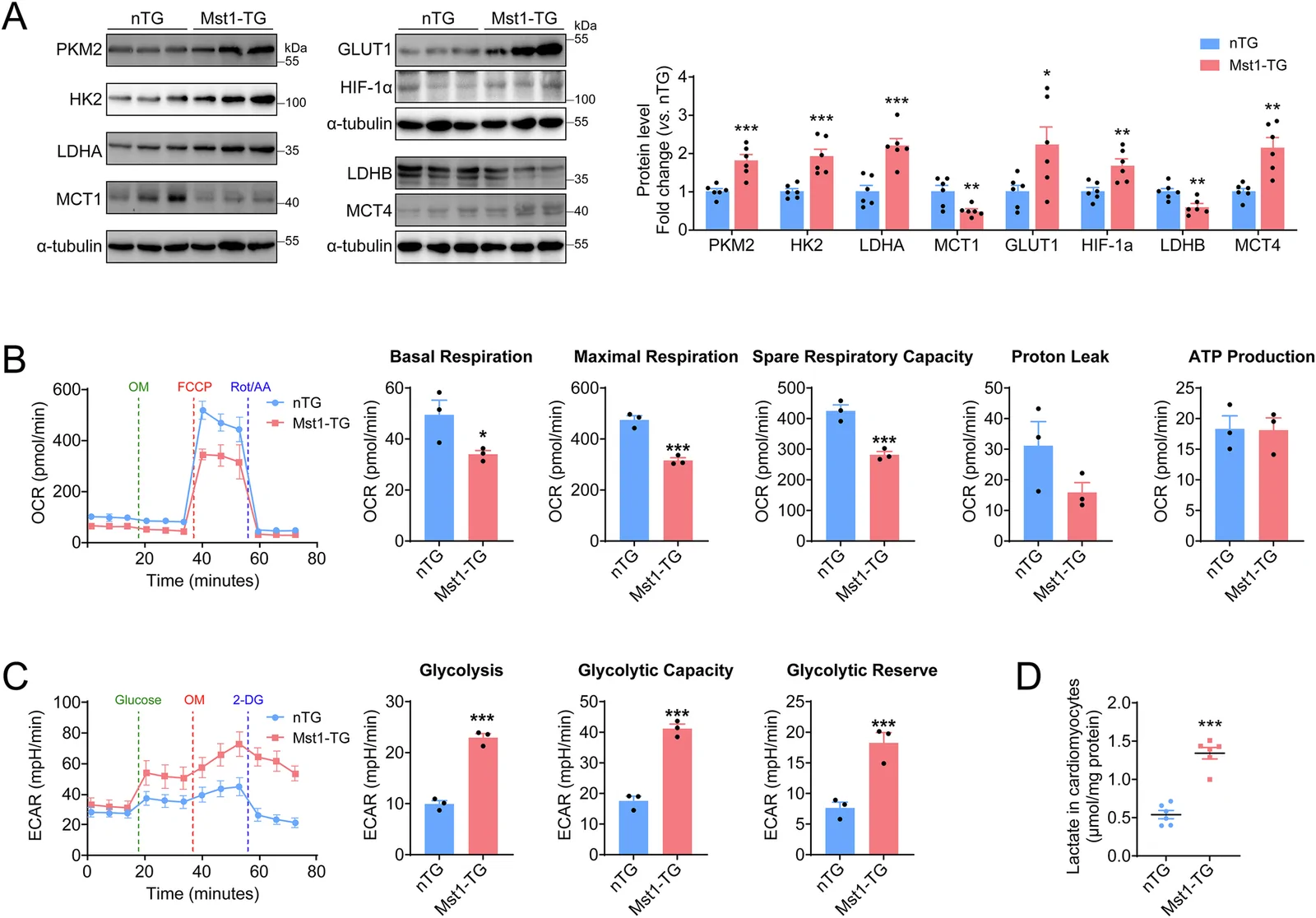
Glycolytic metabolism alterations in isolated adult Mst1-TG mouse cardiomyocytes. **A** Western blotting images and mean values of glycolysis pathway related proteins in the isolated adult mouse cardiomyocytes (*n* = 6). Mitochondrial stress test (**B**) and glycolysis-stress test (**C**) were performed using Seahorse XF96 analyzer on isolated nTG and TG mouse cardiomyocytes (*n* = 3 mice). Oxygen consumption rate (OCR) and extracellular acidification rate (ECAR) values were quantified. **D** Lactate level in isolated cardiomyocytes (*n* = 6). Data are presented as Mean ± SEM, *P < 0.05, **P < 0.01, ***P < 0.001 *vs*. nTG.

To validate the metabolic changes in Mst1-cTG mouse cardiomyocytes, vCMs were isolated from adult nTG and TG mouse hearts via Langendorff perfusion technique. Firstly, we detected the levels of glucose metabolism-related proteins in vCMs by Western blotting. The results showed that protein levels of HIF-1α, glucose transporter GLUT1 and glucose metabolism rate-limiting enzymes PKM2 and HK2 were significantly increased in samples from TG hearts, compared to nTG heart samples (Fig. 2A). In addition, the protein levels for LDHA (catalyzing the conversion of pyruvate to lactate) and the lactate exporter MCT4 increased while levels of LDHB (catalyzing the conversion of lactate to pyruvate) and the lactate importer MCT1 both decreased in samples from the TG group (Fig. 2A). Secondly, using the Seahorse XF analyzer to study freshly isolated vCMs, we evaluated the OCR and ECAR for mitochondrial function or glycolysis. OCR data showed that basal respiration, maximal respiration and spare respiratory capacity were significantly lower in TG relative to nTG vCMs, whereas proton leak and ATP production were unchanged (Fig. 2B). ECAR data showed that basal glycolysis, glycolytic capacity and glycolytic reserve capacity of vCMs were all elevated in TG relative to nTG group (Fig. 2C). These results demonstrated that mitochondrial oxidative metabolism and energy production were impaired while glycolysis was enhanced in TG mouse vCMs. We also measured lactate levels in vCMs and detected a twofold increase in the lactate content of TG compared to nTG samples (Fig. 2D). Thus, these data demonstrated that Hippo pathway activation facilitated cardiomyocyte glycolysis with enhanced lactate production and secretion.

### Fibroblast H3K18la and fibrotic gene expression were both increased in Mst1-cTG mouse myocardium

**Fig. 3:**
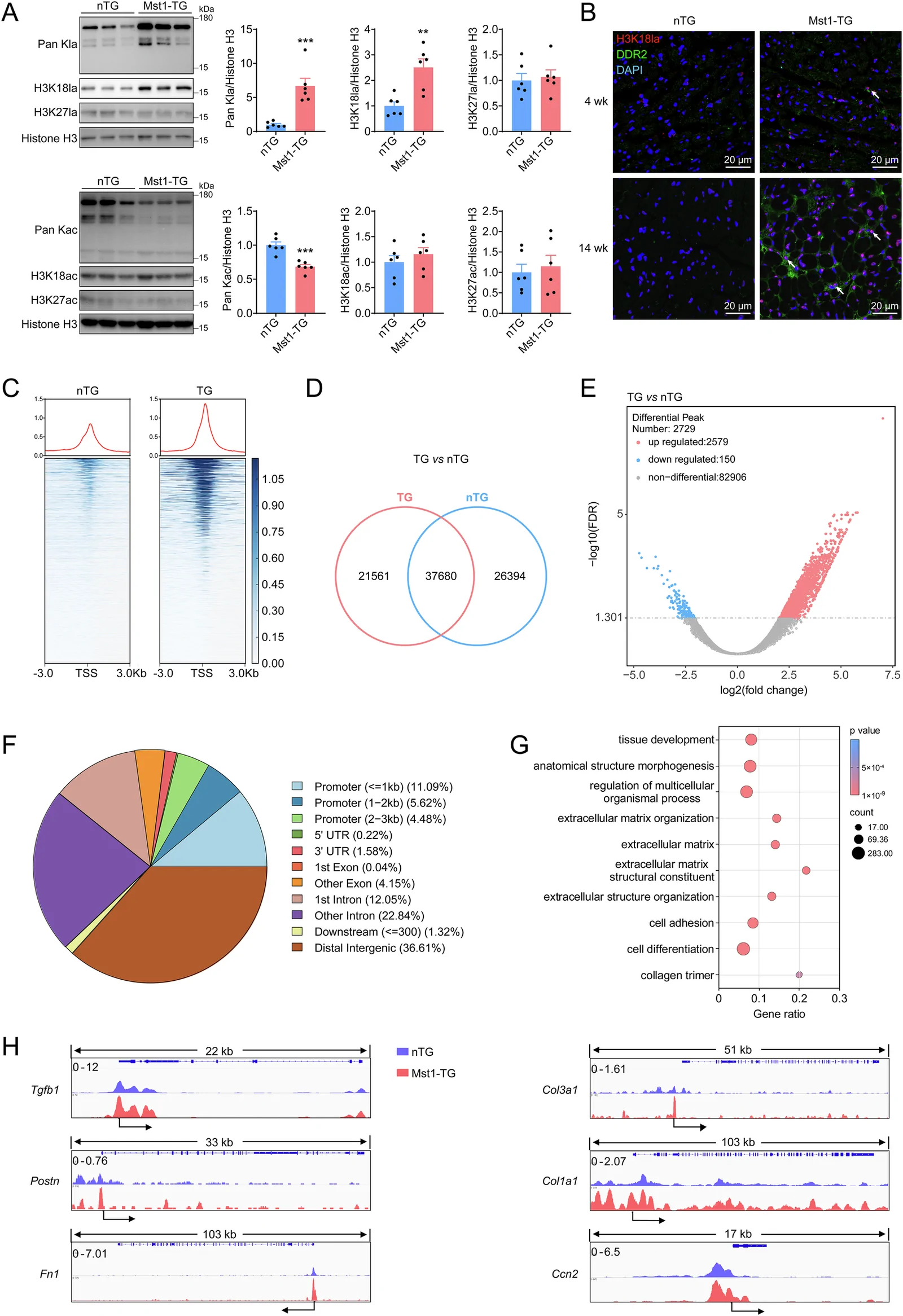
CUT&Tag analysis reveals histone H3K18la regulates fibrosis gene expression in Mst1-TG mouse heart. **A** Western blotting images and mean values of histone lactylation and acetylation level in the LVs. Data are presented as Mean ± SEM, *n* = 6, ^**^P < 0.01, ^***^P < 0.001 *vs*. nTG. **B** Representative immunofluorescent staining of H3K18la (red) and DDR2 (green) in sections of LV tissues from 4-wk and 14-wk old Mst1-TG and nTG mouse hearts, cell nuclei were stained with DAPI (blue). **C** Heatmap showing the genomic occupancy of H3K18la ± 3 kb flanking TSSs in the LVs. The genes shown in rows are sorted in descending order by signal strength. **D** Venn diagram of the number of H3K18la-enriched peaks in nTG and TG mouse LV. **E** Volcano plots showing differential H3K18la- enriched peaks in Mst1-TG mouse heart relative to nTG mice (*n* = 3). **F** Pie chart showing the distribution of H3K18la at annotated genomic regions in the LVs. **G** GO enrichment analysis for upregulated gene sets with increased H3K18la modification (*n* = 3). **H** Normalized read densities for H3K18la at the fibrotic genes.

We studied Mst1-cTG mouse myocardium to identify potential changes in histone lactylation and its regulated downstream genes. Using histone samples extracted from LV tissues, Western blotting results showed elevated levels of histone pan-lysin lactylation (pan-Kla) while pan-lysin acetylation levels decreased in TG mouse LVs. Specifically, H3K18la levels in TG mouse LVs were significantly increased, while H3K18ac levels remained unchanged. H3K27ac has been reported to participate in fibroblast activation and organ fibrosis [32]. However, the abundance of both H3K27la and H3K27ac was unchanged in TG versus nTG LVs (Fig. 3A). Next, we performed immunofluorescence imaging and found that H3K18la immunoreactive signal was more pronounced in LVs of TG versus nTG mice at 4 weeks of age and was further enhanced at 14 weeks of age. Notably, H3K18la immunofluorescence signal was predominantly found in fibroblast nuclei as shown by co-staining with the fibroblast marker DDR2 (Fig. 3B).

We performed CUT&Tag assays to study whether H3K18la might regulate fibrotic gene expression in TG mouse LVs. As shown, we found that H3K18la peaks were increased near transcription start sites (TSSs) in samples from TG mice (Fig. 3C). Using a Venn plot, we detected 64,074 H3K18la enriched peaks in nTG and 59,241 peaks in TG mouse LVs, with 37,680 peaks enriched in the same gene regions of both genotypes (Fig. 3D). Moreover, we detected 2729 differential peaks between both groups. Of them, 2579 peaks were upregulated and 150 downregulated in TG relative to nTG LVs (Fig. 3E). Also, 21% of differential peaks were predominantly mapped to the promoter regions (≤3 kb) of candidate genes (Fig. 3F). GO term analysis revealed that upregulated genes with increased H3K18la enrichment in TG mouse LVs were actively involved in ECM organization, cell differentiation and adhesion (Fig. 3G). In addition, H3K18la peaks in promoter regions of genes associated with fibrosis, including for *Tgfb1*, *Postn*, *Fn1*, *Col1a1*, *Col3a1* and *Ccn2*, were significantly higher in TG versus nTG group (Fig. 3H). Collectively, these results indicate that fibroblast H3K18la enhances profibrotic gene expression and molecular pathways for fibrosis in Mst1-cTG mouse LVs.

### Inhibition of glycolysis or lactate production mitigated cardiac dysfunction and fibrosis in Mst1-cTG mice

**Fig. 4:**
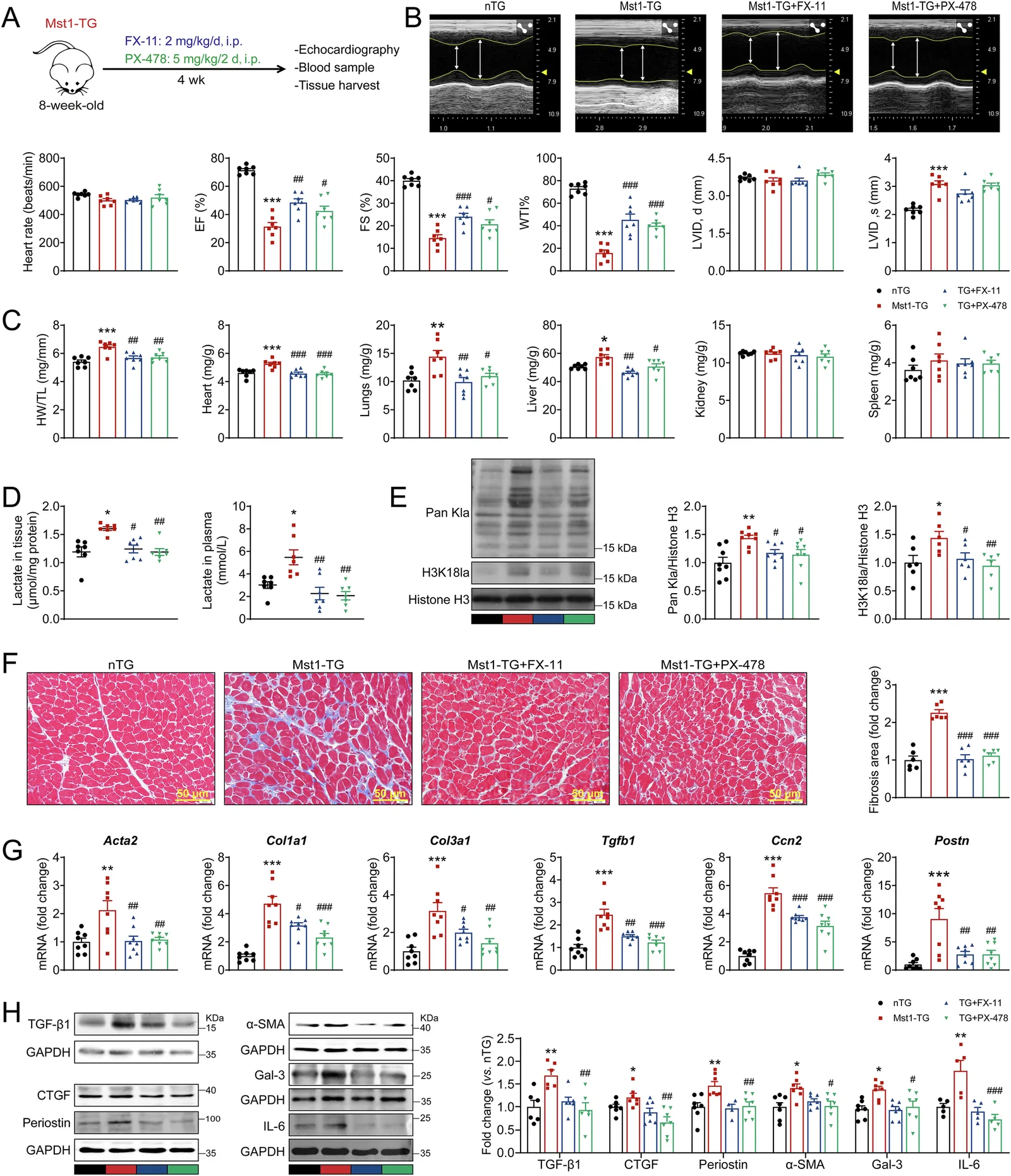
Effects of LDHA or HIF-1α inhibition on fibrosis in Mst1-TG mouse heart. **A** Experimental protocol. Mst1-TG mice received intraperitoneal injection of solvent or LDHA inhibitor (FX-11) or HIF-1α inhibitor (PX-478) for 4 weeks. **B** Representative M-mode tracings from short-axis LV 2-D images and echocardiography derived ejection fraction (EF), fractional shortening (FS), LV internal diameters at end-diastole (LVIDd) and end-systole (LVIDs), wall thickening index (WTI) and heart rate. **C** The ratio of mouse heart weight (HW) to tibia length (TL) and the ratio of mouse organ weight (heart, lung, liver, kidney and spleen) to body weight. **D** Lactate level in LV tissues and plasma. **E** Western blotting images and mean values of histone pan-lactylation and H3K18la level in the LVs. **F** Histological sections of hearts with Masson trichrome staining and quantification of fibrotic area in LVs. **G** Expression of fibrotic genes in LV tissues by RT-qPCR. **H** Western blotting images and mean values of fibrotic protein level in the LVs. Data are presented as Mean ± SEM, *n* = 6-8, ^*^P < 0.05, ^**^P < 0.01, ^***^P < 0.001 *vs*. nTG; ^#^*P* < 0.05, ^##^*P* < 0.01, ^###^*P* < 0.001 *vs*. Mst1-TG.

To demonstrate the causative role of glycolysis and lactate in the development of myocardial fibrosis in Mst1-cTG mice, we treated TG mice with either LDHA inhibitor FX-11 or HIF-1α inhibitor PX-478 for 4 weeks, as depicted in Fig. 4A. Echocardiograph revealed that both inhibitors significantly improved LV systolic function measured as EF, FS and WTI without changes in the heart rate relative to untreated TG mice (Fig. 4B). Moreover, the increased wet weights of the heart, liver and lungs, phenotypes indicative of cardiomegaly and organ congestion, were also improved after FX-11 or PX-478 treatment in TG mice (Fig. 4C). PX-478 treatment efficiently downregulated the expression of glycolytic factors at both mRNA and protein levels (Fig. S2). In addition, FX-11 or PX-478 treatment significantly decreased lactate levels of LV tissue and plasma (Fig. 4D), histone pan-Kla levels and H3K18la levels (Fig. 4E), as well as fibrosis in TG mice. We further examined samples by histology through Masson trichrome staining to find that the excessive interstitial collagen deposition typically observed in TG mouse myocardium was reduced by treatment with FX-11 or PX-478 (Fig. 4F). Moreover, levels of fibrotic genes including *Acta2*, *Col1a1*, *Col3a1*, *Tgfb1*, *Ccn2* and *Postn* (Fig. 4G), as well as levels of proteins including TGF-β1, CTGF, Periostin, α-SMA, Gal-3 and IL-6 (Fig. 4H) in TG mouse LVs were also significantly downregulated in TG mice treated with FX-11 or PX-478. Taken together, these results demonstrate that inhibition of cardiomyocyte glycolysis or lactate production in Mst1-cTG mice downregulates fibroblast H3K18la and mitigates the pathological features of cardiac dysfunction and fibrosis.

### FX-11 treatment ameliorated cardiac dysfunction and fibrosis in mice subjected to IR

**Fig. 5:**
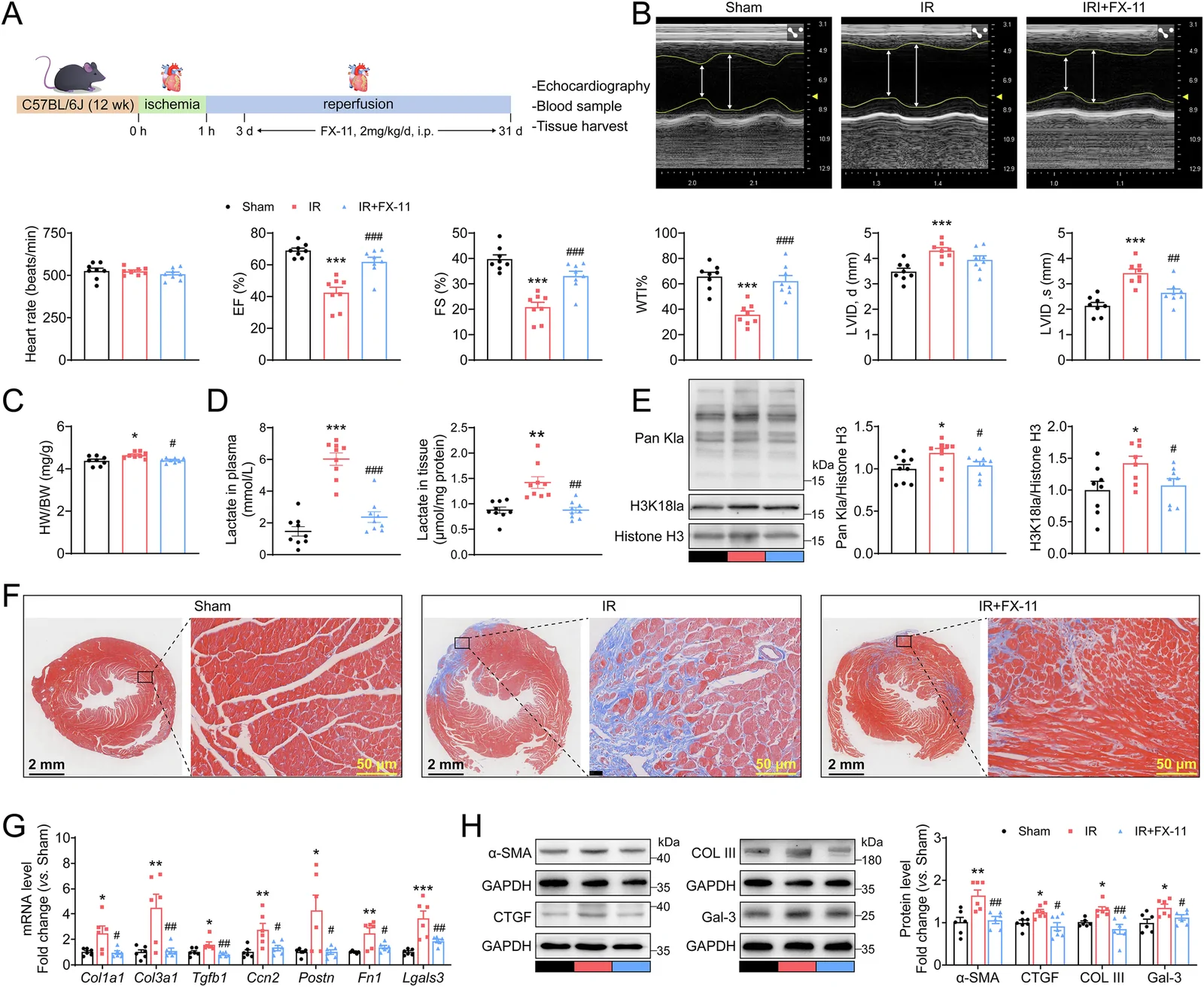
Effects of FX-11 on H3K18la level and fibrosis in ischemia-reperfusion (IR) mouse heart. **A** Experimental protocol. IR mice were received intraperitoneal injection of LDHA inhibitor FX-11 for 28 consecutive days starting from the fourth day after the reperfusion. **B** Representative M-mode tracings from short-axis LV 2-D images and echocardiography derived ejection fraction (EF), fractional shortening (FS), LV internal diameters at end-diastole (LVIDd) and end-systole (LVIDs), wall thickening index (WTI) and heart rate. **C** The ratio of mouse heart weight (HW) to body weight (BW). **D** Lactate level in plasma and LV tissues. **E** Western blotting images and mean values of total histone lactylation and H3K18la level in the LVs. **F** Histological sections of hearts with Masson trichrome staining. **G** Expression of fibrotic genes in LV tissues by RT-qPCR. **H** Western blotting images and mean values of fibrotic protein level in the LVs. Data are presented as Mean ± SEM, *n* = 6-10, ^*^P < 0.05, ^**^P < 0.01, ^***^P < 0.001 *vs*. Sham; ^#^*P* < 0.05, ^##^*P* < 0.01, ^###^*P* < 0.001 *vs*. IR.

For comparison with the Mst1-cTG model, we also studied the IR model to further explore the pro-fibrotic role of lactate and H3K18la in this pathological context. By focusing on the role of fibroblast H3K18la, we tested FX-11 treatment in IR mice commencing from day-4 post IR, so as to not affect the acute inflammatory period post-IR, and then analyzed samples 4 weeks later (Fig. 5A). We found that a 4-week period of treatment with FX-11 significantly improved LV systolic function measured as EF, FS and WTI, and limited LV enlargement without change in the heart rate compared with vehicle-treated IR mice (Fig. 5B). FX-11 treatment not only reversed the increment in the heart weight, as well as the elevated lactate levels of LVs and plasma, but also abolished the increased histone pan-Kla levels and H3K18la levels in IR mice (Fig. 5C–E). Furthermore, augmented collagen deposition in injured areas, as well as the expression of fibrotic genes and proteins in IR mice were significantly attenuated by FX-11 treatment (Fig. 5F–H).

### Fibroblast p300 knockdown reduced H3K18la level and ameliorated cardiac dysfunction and fibrosis in Mst1-cTG mice

**Fig. 6:**
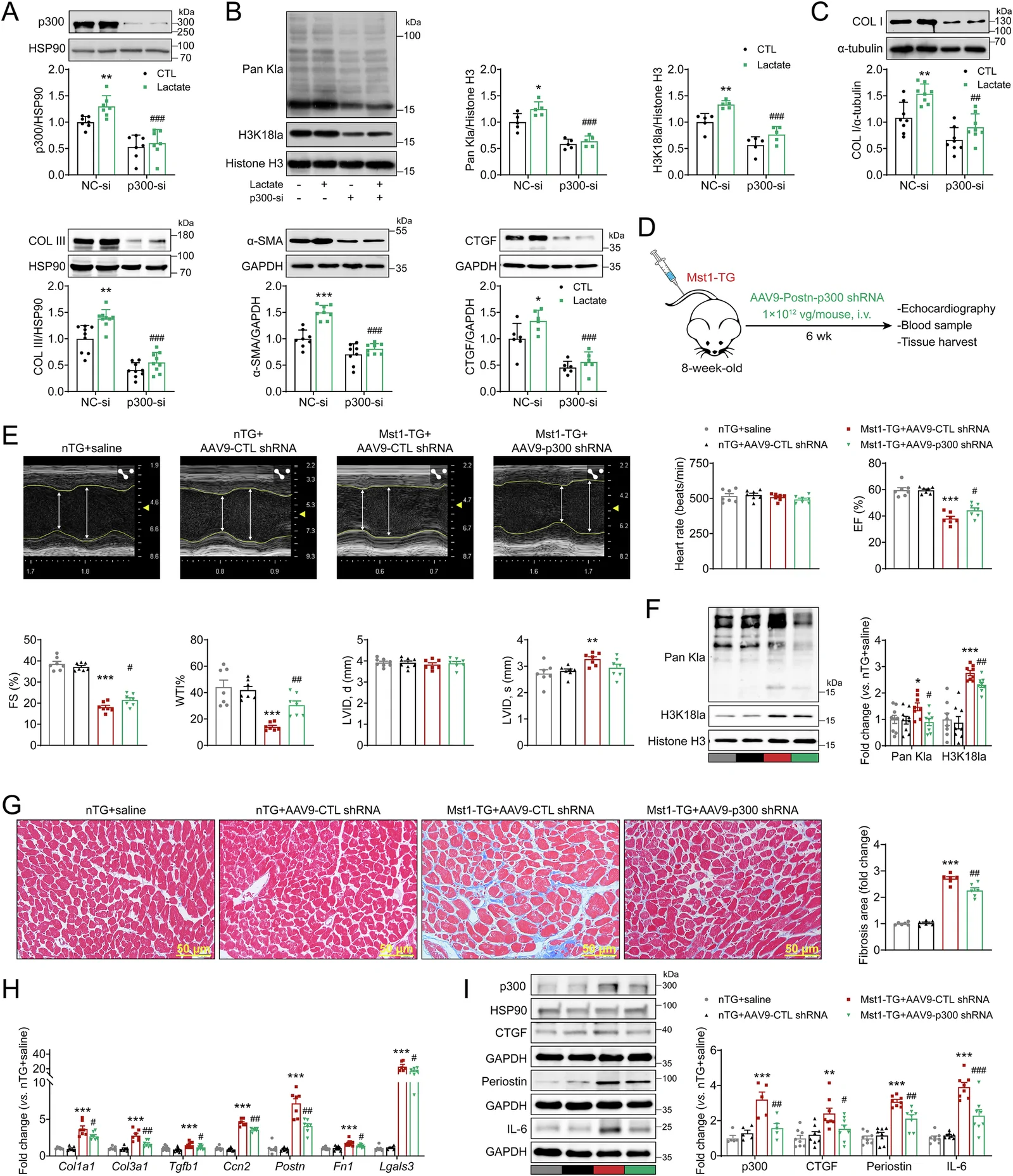
Effects of fibroblast p300 gene knockdown on H3K18la level and fibrosis in Mst1-TG mouse heart. **A**–**C** Primary adult mouse heart fibroblasts were transfected with negative control siRNA (NC-siRNA) or p300-siRNA and then treated with lactate (5 mM, 48 h) or vehicle. **A**–**C** Western blotting images and mean values of p300 protein level, histone lactylation level and fibrotic protein level in fibroblasts. Data are presented as Mean ± SEM, *n* = 5-8, ^*^P < 0.05, ^**^P < 0.01, ^***^P < 0.001 *vs*. NC-siRNA^; ##^*P* < 0.01, ^###^*P* < 0.001 *vs*. lactate+NC-siRNA. **D** Experimental protocol. 8-week-old nTG and Mst1-TG mice injected with saline or 5 × 10^11^ v.g. AAV9-postn-CTL shRNA or AAV9-postn-p300 shRNA through tail vein. After 6 weeks, mice were sacrificed. **E** Representative M-mode tracings from short-axis LV 2-D images and echocardiography derived ejection fraction (EF), fractional shortening (FS), LV internal diameters at end-diastole (LVIDd) and end-systole (LVIDs), wall thickening index (WTI) and heart rate. **F** Western blotting images and mean values of histone pan-lactylation and H3K18la level in the LVs. **G** Histological sections of hearts with Masson trichrome staining and quantification of fibrotic area in LVs. **H** Expression of fibrotic genes in LV tissues by RT-qPCR. **I** Western blotting images and mean values of fibrotic protein level in the LVs. Data are presented as Mean ± SEM, *n* = 5-8, ^*^P < 0.05, ^**^P < 0.01, ^***^P < 0.001 *vs*. nTG+AAV9-CTL shRNA; ^#^*P* < 0.05, ^##^*P* < 0.01, ^###^*P* < 0.001 *vs*. Mst1-TG + AAV9-CTL shRNA.

The acyltransferase p300 is known to function as lactylation “writer” [33]. To investigate the possibility that p300 might regulate lactate-mediated H3K18la levels in fibroblasts, p300 siRNAs and AAV9-postn-p300 shRNAs were constructed to achieve fibroblast-restricted p300 knockdown in vitro or in vivo. In cultured fibroblasts, 5 mM concentration of lactate was selected to stimulate cells according to the results of cell viability and CTGF protein expression with the use of different concentrations of lactate (Fig. S3A, B). Lactate significantly increased levels of histone pan-Kla and H3K18la, and fibrotic protein expression including collagen (COL) I and III, α-SMA and CTGF. Treatment with p300 siRNA in fibroblasts reduced p300 protein expression by approximately 50%, an effect associated with reversal of lactate-induced upregulation of histone lactylation and fibrotic marker proteins (Fig. 6A–C).

We next performed experiments in which AAV9-postn-p300 shRNA was used to knockdown fibroblast p300 in Mst1-cTG hearts in vivo, as depicted in Fig. 6D. At week-6 after tail-vein injection of the virus, AAV9-postn-p300 shRNA-treated TG mice showed a 55% decrease in the myocardial p300 expression relative to TG mice receiving AAV9-postn-CTL shRNA (Fig. 6I), and this result is consistent with immunofluorescence detection of p300 protein signals (Fig. S4). Notably, virally-mediated fibroblast-targeted p300 knockdown in Mst1-cTG mice was associated with significant improvements in LV function and HF phenotype, indicated by improved EF, FS and WTI, and reductions in LVIDs as well as reduced wet weights of the heart, lungs and liver relative to CTL-virus-treated TG mice (Figs. 6E and S5). Moreover, myocardial levels of histone pan-Kla and H3K18la (Figs. 6F and S6), as well as the severity of myocardial fibrosis, estimated by quantitative histology and expression of fibrotic markers at mRNA and protein levels, were all significantly decreased in TG mice treated with AAV9-postn-p300 shRNA versus control treatment (Fig. 6G–I). There was no significant difference in H3K9ac levels and H3K27ac levels in myocardium samples among groups (Fig. S7). These results demonstrated that fibroblast p300 knockdown mitigated cardiac dysfunction and fibrosis via suppressing lactate-mediated H3K18la.

### H3K18la promoted fibrotic gene transcription in synergy with transcription factor RUNX2

**Fig. 7:**
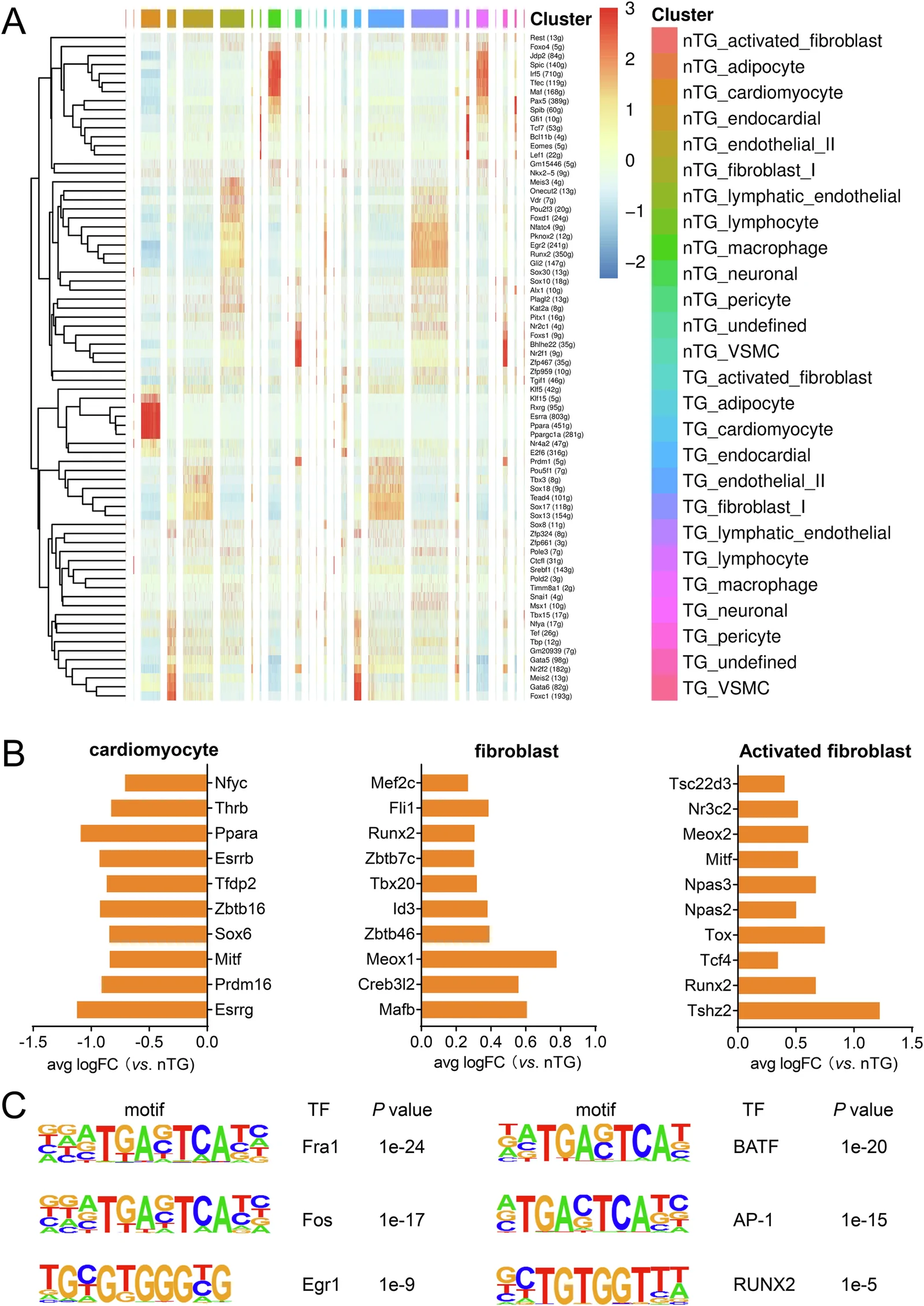
Combination of snRNA-seq and CUT&Tag analysis identifies the transcription factors driving fibrogenesis in Mst1-TG heart. **A** Heat map showing top-5 activity altered transcription factors in each cell cluster of Mst1-TG mouse heart relative to nTG mice from SnRNA-seq transcription factor activity analysis. **B** Top-10 expression changed transcription factors in cardiomyocyte, fibroblast and activated fibroblast cell clusters of Mst1-TG mouse heart relative to nTG mice. **C** Motif analysis of CUT&Tag data showing the potential transcription factors binding to H3K18la enriched gene regions in Mst1-TG mouse heart.

We investigated the top-5 most active TFs of 13 major cell types in TG versus nTG hearts according to TF activity analysis in the snRNA-seq database (Fig. 7A). In TG mouse cardiomyocytes, Esrra, Ppara and Ppargc1a were TFs that were the most significantly inactivated relative to nTG. In cardiac fibroblasts and activated fibroblasts of TG mice, the activities of a few TFs were enhanced relative to nTG, especially Runx2 and Egr2. The expression of top-10 changed TFs in TG mouse cardiomyocytes was all decreased compared to nTG, including Esrr and Ppar families. In fibroblasts and activated fibroblasts of TG mice, the expression levels for the top-10 significantly altered TFs were all increased, with expression of Runx2 upregulated in cell clusters, and also more significantly in activated fibroblasts (Fig. 7B). These data indicated that suppressed OXPHOS levels in cardiomyocytes of TG mice arose through a mechanism that involved the downregulated TFs including Esrr and Ppar families (Fig. 7A, B), as well as the possibility that TFs with upregulated activity and expression in fibroblasts, especially Runx2, might be relevant to fibroblast activation in TG hearts. By motif analysis using H3K18la CUT&Tag data, potential TFs that regulated fibrotic gene transcription in synergy with H3K18la were identified, including Fra1, BATF, Fos1, AP-1, Egr1 and RUNX2 (Fig. 7C). Taken together, our results suggest that RUNX2 is a potential TF promoting fibrotic gene expression with H3K18la in fibroblasts of Mst1-cTG mice.

### RUNX2 inhibition mitigated cardiac fibrosis in Mst1-cTG mice

**Fig. 8:**
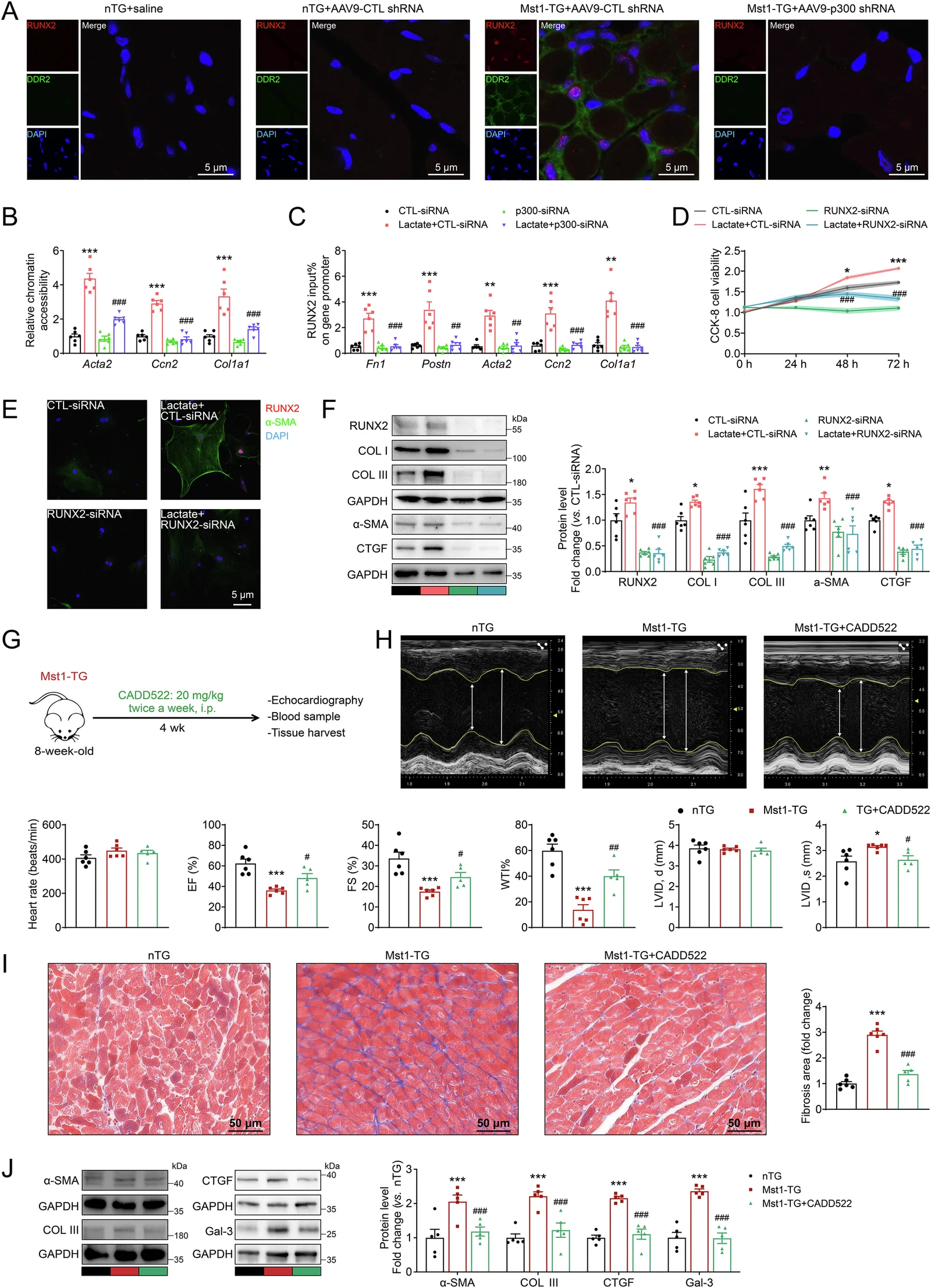
Effects of RUNX2 inhibition on fibrosis in Mst1-TG mouse heart. **A** Representative immunofluorescent staining of RUNX2 (red) and DDR2 (green) in sections of mouse LV tissues; cell nuclei were stained with DAPI (blue). In **B** and **C**, primary adult mouse heart fibroblasts were transfected with control siRNA (CTL-siRNA) or p300-siRNA and then treated with lactate (5 mM) or vehicle. **B** RT-qPCR analysis of chromatin accessibility at the fibrotic gene promoter (*n* = 6). **C** Enrichment for RUNX2 at the fibrotic gene promoter was assessed by RT-qPCR after CUT&RUN assay. *n* = 6, ^**^P < 0.01, ^***^P < 0.001 *vs*. CTL-siRNA; ^##^*P* < 0.01, ^###^*P* < 0.001 *vs*^.^ lactate+CTL-siRNA. In **D**–**F**, primary adult mouse heart fibroblasts were transfected with CTL-siRNA or RUNX2-siRNA and then treated with lactate (5 mM) or vehicle. **D** Cell viability tested by CCK-8 kit at different time points after siRNA transfection and/or following lactate stimulation (*n* = 12). **E** Representative immunofluorescent staining of RUNX2 (red) and α-SMA (green) in fibroblasts; cell nuclei were stained with DAPI (blue). **F** Western blotting images and mean values of RUNX2 and fibrotic protein level in fibroblasts. *n* = 6, ^*^P < 0.05, ^**^P < 0.01, ^***^P < 0.001 *vs*. CTL-siRNA; ^###^*P* < 0.001 *vs*. lactate+CTL-siRNA. **G** Experimental protocol. Mst1-TG mice injected (i.p.) with solvent or RUNX2 inhibitor (CADD522) twice a week for 4 weeks. **H** Representative echocardiographic M-mode tracings from short-axis LV 2-D images and derived ejection fraction (EF), fractional shortening (FS), LV internal diameters at end-diastole (LVIDd) and end-systole (LVIDs), wall thickening index (WTI) and heart rate (*n* = 5–6). **I** Histological sections of hearts with Masson-trichrome staining and quantification of fibrotic area in LVs (*n* = 5–6). **J** Western blotting images and mean values of fibrotic protein level in the LVs. *n* = 5–6, ^*^P < 0.05, ^***^P < 0.001 *vs*. nTG; ^#^*P* < 0.05, ^##^*P* < 0.01, ^###^*P* < 0.001 *vs*. Mst1-TG. Data are presented as Mean ± SEM.

The role of RUNX2 in lactate-mediated fibroblast activation was further studied. Immunofluorescent staining of Mst1-cTG mouse LV sections revealed that fibroblast p300 knockdown via AAV9-postn-p300 shRNA also downregulated RUNX2 levels in fibroblast nuclei, apart from reducing H3K18la levels compared to TG mice that received CTL-virus (Fig. 8A). In cultured cardiac fibroblasts, p300 gene silencing effectively reversed lactate-induced increase in both chromatin accessibility and RUNX2 enrichment at promoters of selected fibrotic genes (Fig. 8B, C). In addition, RUNX2-siRNA reduced RUNX2 protein levels by 70% compared to CTL-siRNA treated cells (Fig. 8F) and significantly inhibited lactate-induced fibroblast proliferation measured by a CCK8 assay (Fig. 8D). Also, we performed immunofluorescence studies with cultured fibroblasts and found that lactate significantly enhanced levels of nuclear RUNX2 and expression of the myofibroblast marker α-SMA, while treatment with RUNX2-siRNA abolished these effects of lactate treatment of these cells (Fig. 8E). Importantly, RUNX2 knockdown inhibited lactate-induced upregulation of fibrotic proteins, including α-SMA, COL I and III and CTGF in cultured fibroblasts (Fig. 8F).

At last, we investigated the effect of RUNX2 inhibition on cardiac fibrosis in vivo by treating Mst1-cTG mice for 4 weeks with the RUNX2 inhibitor CADD522. Such treatment significantly reversed cardiac systolic function measured as EF, FS and WTI, and attenuated chamber dilation (Fig. 8H). Fibrotic phenotypes in TG mouse hearts, including increased collagen deposition quantified by histology and upregulated fibrotic protein expression, were also significantly suppressed by CADD522 treatment (Fig. 8I, J). Collectively, these results demonstrate that RUNX2 plays a critical role in lactate-mediated fibroblast activation via facilitating fibrotic gene transcription and that RUNX2 inhibition in vivo can effectively improve cardiac fibrosis.

## Discussion

The current study has made three novel findings. *First*, cardiomyocyte-restricted Hippo pathway activation induces metabolic disorder together with lactate overproduction, that acting as a signal molecule to activate fibroblasts and the fibrotic process. Inhibition of either glycolysis or lactate production attenuates myocardial fibrosis in Mst1-cTG or myocardial IR models. *Second*, lactate-mediated fibroblast H3K18la epigenetically enhances fibrotic gene expression, while knockdown of fibroblast p300, the “writer” of H3K18la, decreases H3K18la levels and attenuates fibrosis. *Third*, the transcription factor RUNX2 cooperates with H3K18la to upregulate fibrotic gene transcription in cardiac fibroblasts. RUNX2 inhibition in vivo also inhibited myocardial fibrosis in Mst1-cTG mice.

The Hippo pathway was proven to be activated in cardiac biopsy samples from human patients with ischemic or non-ischemic cardiomyopathy, HF or arrhythmic cardiomyopathy [8, 12, 34]. Experimentally, there has been strong evidence that myocardial Hippo pathway activation [6, 35] or inactivation of YAP/TEAD1 [7, 11, 12] leads to DCM with mitochondrial damage and metabolic disorder, while inactivation of the myocardial Hippo pathway is cardioprotective in anti-cancer drug-induced cardiotoxicity or Takotsubo syndrome [6, 10, 36]. In the Mst1-cTG model with cardiomyocyte-restricted Hippo pathway activation, TG mice exhibited obvious DCM from 3 weeks of age, with cardiac dysfunction and interstitial fibrosis [6]. Likewise, it has been reported that YAP-cKO and TEAD1-cKO mice display rapid onset of DCM phenotypes, interstitial fibrosis and cardiomyocyte metabolic remodeling [7, 11, 12]. In the current study, the cellular basis for DCM in Mst1-cTG mice was characterized via snRNA-seq of cardiomyocytes, which revealed significant molecular signatures of metabolic disorder, including suppressed OXPHOS, FAO and BCAA catabolism pathways, together with enhanced HIF-1α-driven glycolysis, all of which are consistent with our previous reports [6, 21]. Particularly, our snRNA-seq studies further suggested that fibroblast activation is a unique cellular feature of DCM that would potentiate the effects of upregulated fibrotic genes. These findings altogether indicate that Hippo pathway-induced metabolic remodeling in cardiomyocytes causes fibroblast activation and fibrosis, leading to cardiomyopathy.

Using a model of cardiomyocyte-specific Hippo pathway inhibition by transgenic expression of mutant dominant-negative Mst1 gene (dnMst1-TG), we previously found that under conditions of cardiac Hippo pathway activation induced by doxorubicin or isoprenaline, the concomitant elevation of lactate levels in LV tissue and plasma was largely abolished in dnMst1-TG versus nTG mice [10, 36]. Here, using isolated cardiomyocytes from adult Mst1-cTG mouse LV, we provided further strong evidence for the enhanced glycolysis and lactate production as a consequence of Hippo pathway activation in cardiomyocytes. Given the existing clinical evidence of enhanced cardiac Hippo signaling pathway in the settings of cardiomyopathy and HF [37, 38], the cell-specific metabolic remodeling, as shown in Mst1-cTG mice, represents an ideal model to explore the link between cardiomyocytes and fibroblasts via metabolites, in particular cardiomyocyte-derived lactate.

Lactate-mediated protein lactylation has been shown to regulate multiple biological processes including immune response mode, cell differentiation and tumor progression [39–41]. Recent studies have revealed the role of protein lactylation in the onset of myocardial fibrosis. In mice subjected to MI, H3K18la of monocytes facilitated reparative gene expression to promote cardiac repair processes, while Snail1 lactylation in endothelium facilitated EndoMT [18, 19]. In addition, in the failing myocardium of mice or human subjects, excessive lactate export from cardiomyocytes via MCT4 reduced the extent of α-MHC lactylation, and this impaired cardiac function through disruption of the structural integrity of sarcomeres with titin [20]. Nevertheless, the effects of cardiomyocyte-derived lactate on fibroblasts and fibrosis had not been reported, and our current study now demonstrates that, in the context of activated Hippo pathway signaling, cardiomyocyte-derived lactate can induce fibroblast H3K18la in the presence of the acetyltransferases p300, leading to fibroblast activation, upregulation of fibrotic genes and myocardial fibrosis. In Mst1-cTG mouse hearts, histone pan-Kla levels and H3K18la levels significantly increased, relative to histone acetylation and lactylation of other specific sites. Our CUT&Tag results indicated that H3K18la was predominantly detected in fibroblasts and enriched in the promoter regions of multiple fibrotic genes that were associated with enhanced transcription. Our in vitro experiments also proved that lactate promoted fibroblast proliferation and activation via elevating H3K18la levels. These findings clarify a pivotal role of fibroblast H3K18la in activating the fibrotic gene profile and onset of fibrosis. Latest studies have also reported increased level of H3K18la in cardiac tissues of heart failure patients with atrial fibrillation [42], hypertrophy [43] or diabetic cardiomyopathy [44]. Collectively, myocardial H3K18la is a potential therapeutic target for heart diseases.

In vivo inhibition of glycolysis with HIF-1α inhibitor PX-478 or of lactate production with LDHA inhibitor FX-11 in Mst1-cTG mice was found to be beneficial in ameliorating cardiac dysfunction and fibrosis, and the efficacy of these treatments was accompanied by the downregulation of fibroblast H3K18la levels. PX-478 is a small-molecule HIF-1α inhibitor that reduces HIF-1α protein levels by inhibiting its mRNA expression and translation, and also by increasing HIF-1α degradation via the ubiquitin-proteasome pathway in cells [45]. Previous studies demonstrated the efficacy of PX-478 as a monotherapy or in combination with other agents in breast cancer and hepatocellular carcinoma, and phase I clinical trials on advanced solid tumors or lymphoma have been completed with safe oral tolerance and effective HIF-1α inhibition [46, 47]. FX-11 is a potent, selective, reversible and competitive LDHA inhibitor that also displays antitumor activity in lymphoma and pancreatic cancer xenografts [24, 48]. Our study validated the therapeutic efficacy of both drugs on myocardial fibrosis and revealed that each influenced metabolic and epigenetic changes, suggesting the potential of these anti-cancer drugs for the treatment of heart disease. Moreover, our AAV9-mediated fibroblast p300 knockdown studies using Mst1-cTG mice were notable for their efficiency in downregulating fibroblast H3K18la and mitigating LV fibrosis and dysfunction. Interestingly, p300 disruption exerts no effect on acetylation levels of H3K9 and H3K27, which are also known to be affected by activities of other acetyltransferases or deacetylases. Thus, apart from glycolysis or LDHA antagonism, p300 may be a relevant therapeutic target for cardiac fibrosis through a mechanism that involves reductions to H3K18la levels in fibroblasts.

To verify our findings in the Mst1-cTG model regarding lactate-induced fibroblast H3K18la and fibrosis in alternative diseased conditions, we studied the IR model that is known to be accompanied by activation of the myocardial Hippo pathway and later on fibrosis [49]. Specifically, we investigated the changes in lactate content and histone lactylation, and tested the efficacy of FX-11 on cardiac fibrosis and dysfunction. While it is known that Mst1-cTG mice with cardiomyocyte-specific Mst1 overexpression feature minimal inflammatory infiltration in the myocardium [21, 50], the mechanism of myocardial fibrosis in the IR model is more complex and involves ischemic cardiomyocyte death, oxidative stress, microvascular injury and leakage as well as severe inflammation [23, 51]. Of note, intense inflammatory cell infiltration and massive release of inflammatory cytokines after IR are features that are implicated as events that precede fibrotic healing [52]. Furthermore, H3K18la levels in monocytes have been implicated in regulating expression of reparative genes at the early stage after MI [18]. Hence, to avoid interfering with inflammatory cell lactylation in the acute phase while focusing on fibroblasts, FX-11 was administered from day-4 post-IR, when the inflammatory peak in response to IR has ostensibly subsided [52]. Whilst the cellular source of lactate in Mst1-TG mice is primarily derived from cardiomyocytes, the situation in the IR model is more complex, with lactate generated by diverse cell types including activated fibroblasts, endothelial cells and remaining macrophages (M2). Nevertheless, treatment with FX-11 in mice subjected to IR was also effective in lowering tissue and plasma contents of lactate, reducing the degree of H3K18la, LV dysfunction, HF phenotypes, and cardiac fibrosis. Thus, H3K18la-mediated fibroblast activation is a common mechanism of cardiac fibrosis.

Upregulation of fibrotic gene expression in fibroblasts driven by H3K18la requires TFs to bind to and activate the promoters of fibrotic genes. Yet, H3K18la-associated TFs in the setting of fibrogenesis remain underexplored. Using snRNA-seq and the CUT&Tag database of the Mst1-cTG mouse heart, we have described the activities of TFs in regulating fibrotic gene expression together with fibroblast H3K18la. In fibroblasts and activated fibroblasts of TG hearts, the activities of Runx2, Nfatc4, Pknox2, Egr2 and Gli2 were most significantly increased, and Runx2 was the second-highest-expressed TF. In addition, motif analysis using H3K18la CUT&Tag database detected with high probability that Runx2 bound to H3K18la-enriched promoter regions in TG hearts. In fibroblasts in vitro, we also revealed RUNX2 was enriched to H3K18la-opened chromatin at fibrotic gene promoters with enhanced transcription of fibrotic genes. Previous studies showed that RUNX2 acts as a key TF promoting pulmonary and hepatic fibrosis [53, 54]. Here, the anti-fibrotic property of RUNX2 inhibitor CADD522 is observed in Mst1-cTG mouse hearts. Altogether, RUNX2 was the critical TF responding to H3K18la to activate fibrotic genes in cardiac fibroblasts and this clarifies the putative transcriptional mechanisms following epigenetic modification in cardiac fibrosis.

This study illuminated the important common mechanism of fibrosis in cardiomyopathy with Hippo pathway activation, in which lactate as a signaling molecule, elevates fibroblast H3K18la to activate a fibrotic gene profile. Myocardial metabolic remodeling is implicated as a common event in heart disease [4, 55]. In line with our findings from the Mst1-cTG model, numerous experimental studies reported that cardiomyocyte-restricted genetic interventions of contractile or structural proteins resulted in cardiac phenotypes that exhibit not only cardiomegaly and dysfunction, but also severe fibrosis [56–61]. Similarly, cardiac fibrosis constitutes an important histopathology in patients with DCM due to genetic mutations [62]. Given the existing preclinical and clinical evidence for Hippo pathway activation that causatively results in metabolic remodeling and cardiomyopathy [6–8, 12, 34], further preclinical and clinical studies are warranted to validate this mechanism in a broader setting of heart diseases and to explore therapies for cardiac fibrosis through intervening metabolic remodeling and cardiomyocyte-derived lactate overproduction, as illustrated in the current study. One limitation in our study that deserves discussion is that we mainly focused on the role of fibroblast H3K18la in fibrosis without determining other specific sites, albeit total lactylation level was determined. Although H3K18la has been reported to play pivotal roles in liver and heart fibrosis [16, 18, 42], it remains to be determined whether other histone lactylation site(s) form a therapeutic target against organ fibrosis. Future studies employing mass spectrometry would be helpful to solve this question.

In conclusion, we demonstrate that myocardial Hippo pathway activation promotes production and release of lactate that acts in a paracrine fashion to activate fibroblasts through upregulating H3K18la and RUNX2 recruitment to the promoter regions of fibrotic genes. Blockade of excess lactate production via inhibiting HIF-1α or LDHA, fibroblast p300 knockdown to inhibit histone lactylation, or suppression of RUNX2 transcriptional activity all efficiently attenuate fibrosis in Mst1-cTG and IR models. Our findings clarify the epigenetic and transcriptional mechanisms of fibrosis driven by cardiomyocyte Hippo pathway activation and provide insights into cardiomyocyte-fibroblast metabolic coupling leading to fibrosis.

## Supplementary information

Supplemental Tables and Figures

Uncropped Western Blots

## Data Availability

The study’s data can be obtained from the corresponding author upon reasonable request.
