# Supplementary material for: Inhibition of lactate-histone lactylation mediated cardiomyocyte-fibroblast communication mitigates cardiac fibrosis in cardiomyopathy models with Hippo pathway activation
Source: Cell Death Dis. 2026 Aug 1;17(1):922. doi: 10.1038/s41419-026-09137-7 (PMC13720063; doi:10.1038/s41419-026-09137-7)
Supplement: Supplementary file 1 [file 41419_2026_9137_MOESM2_ESM.pdf]

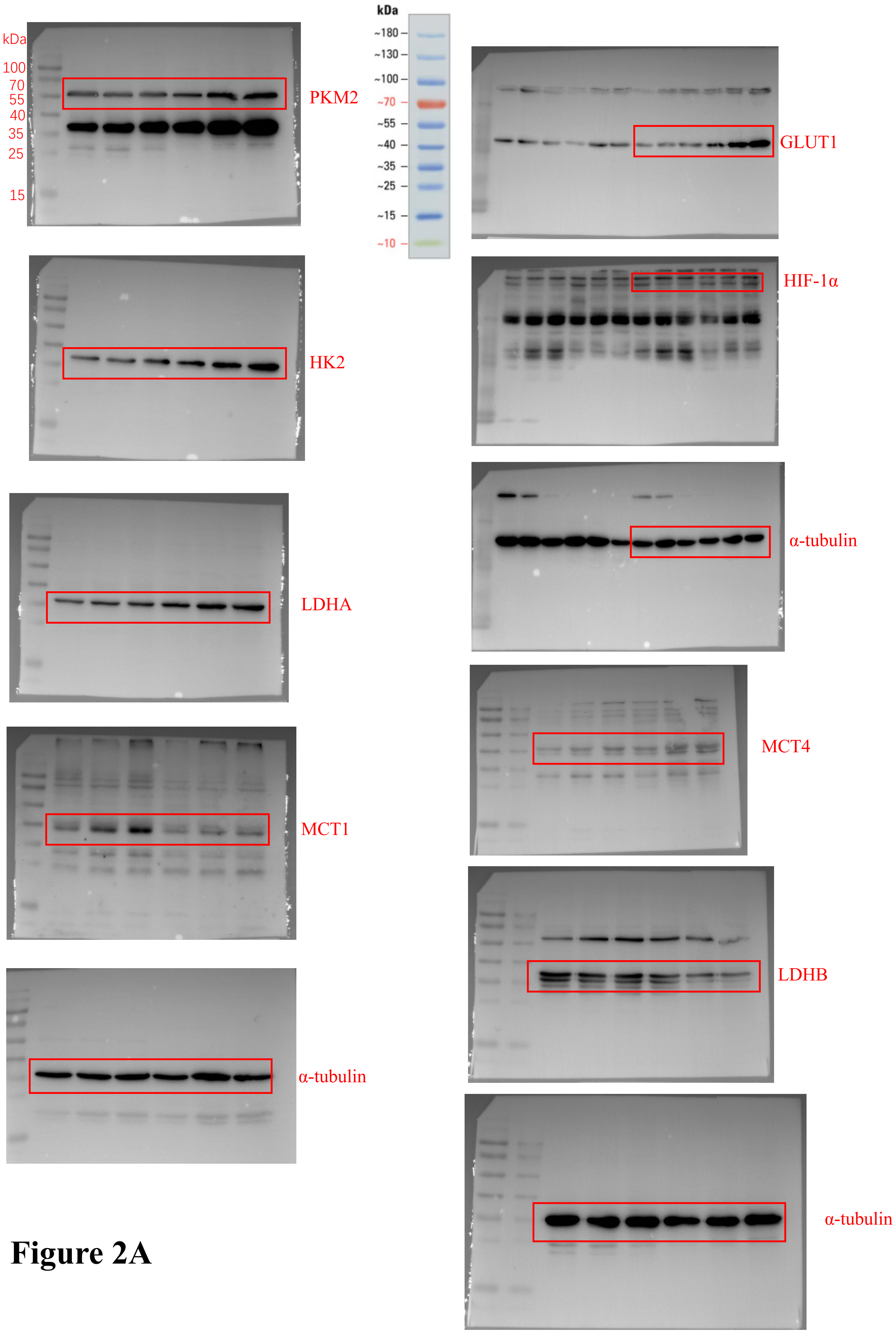
kDa
100
70
55
40
35
25
15
PKM2
HK2
LDHA
MCT1
α-tubulin
kDa
~180
~130
~100
~70
~55
~40
~35
~25
~15
~10
GLUT1
HIF-1α
α-tubulin
MCT4
LDHB
α-tubulin

Figure 2A

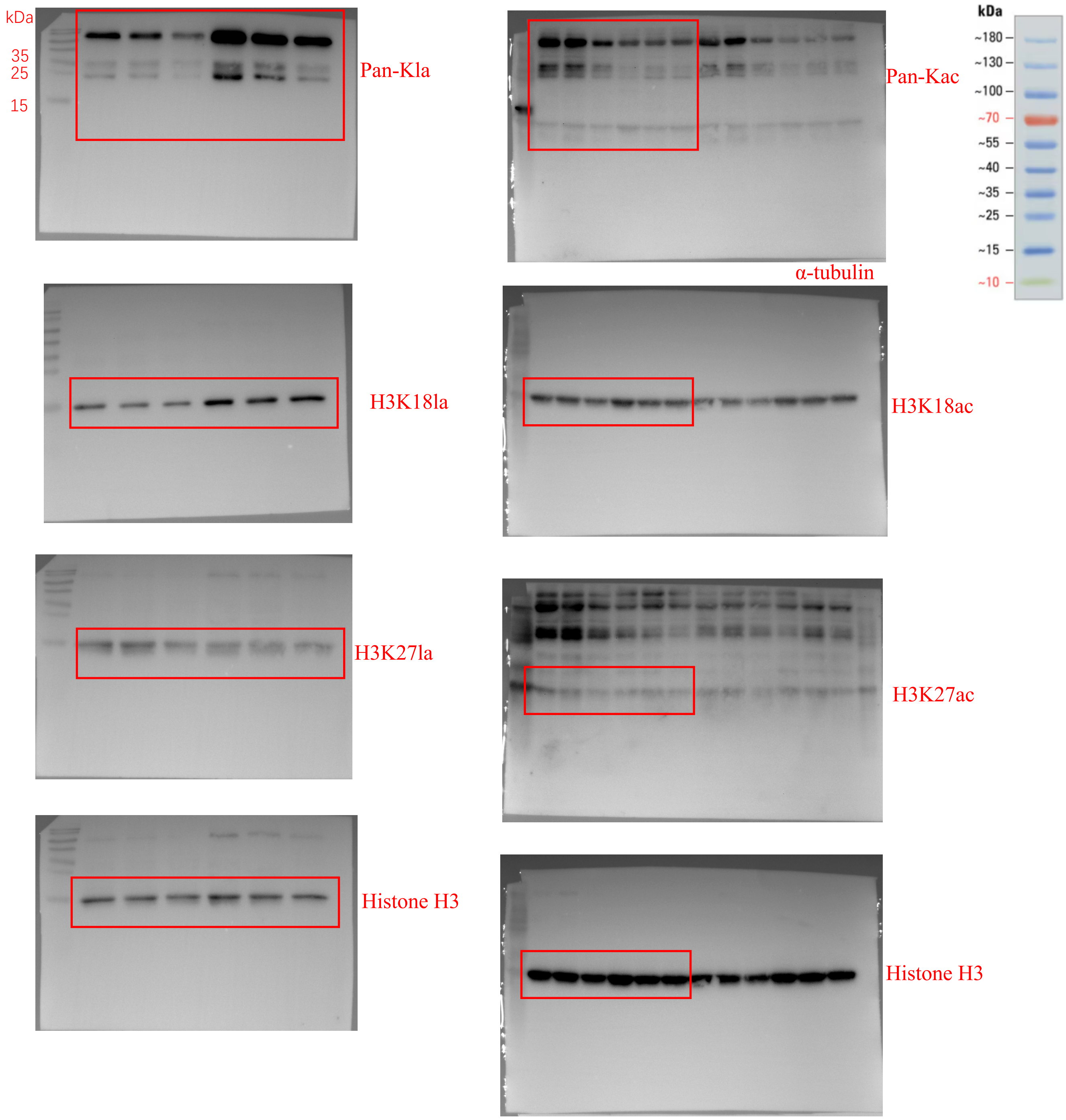
kDa
35
25
15
Pan-Kla
Pan-Kac
kDa
~180
~130
~100
~70
~55
~40
~35
~25
~15
~10
α-tubulin
H3K18la
H3K18ac
H3K27la
H3K27ac
Histone H3
Histone H3

Figure 3A

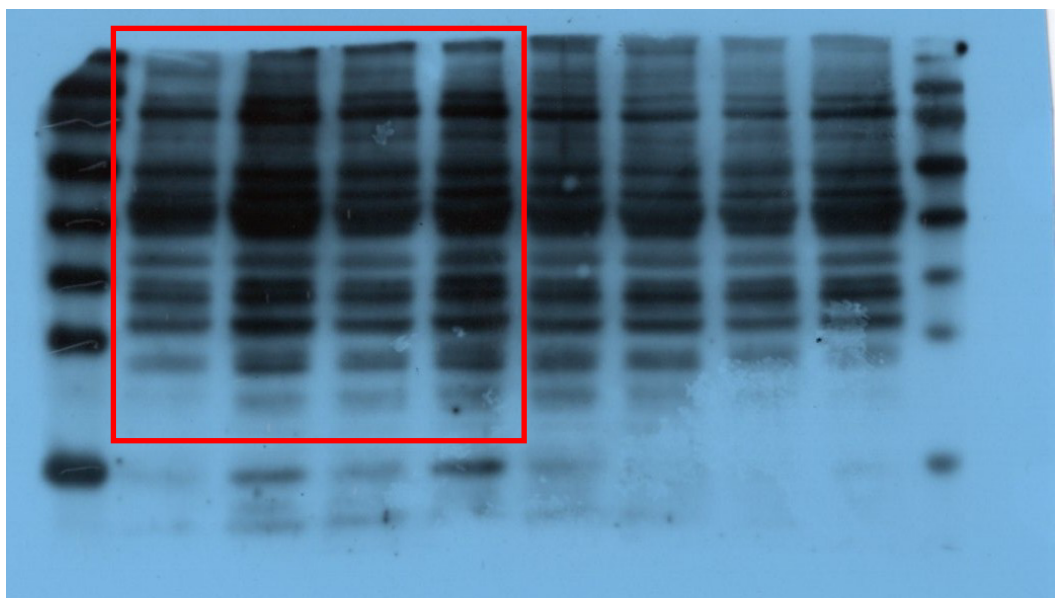

Pan-Kla

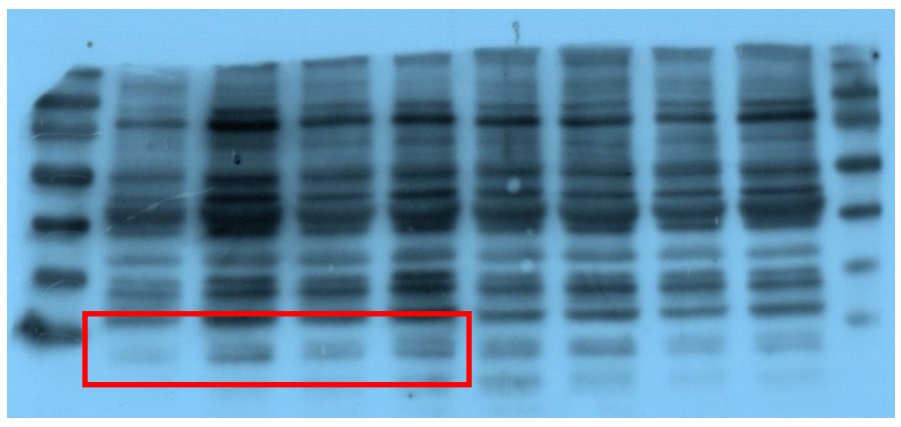

H3K18la

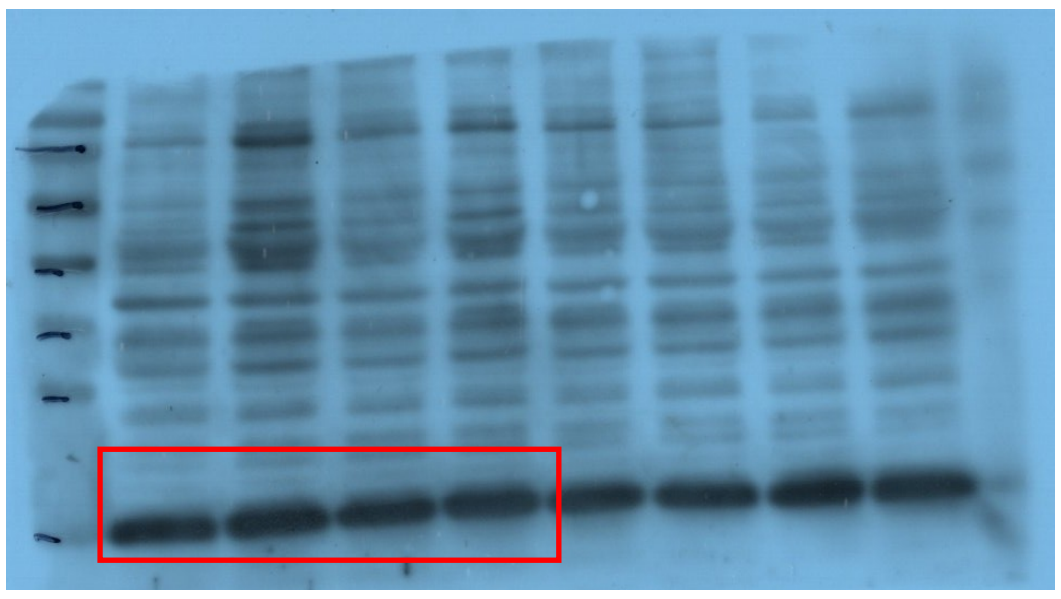

Histone H3

Figure 4E

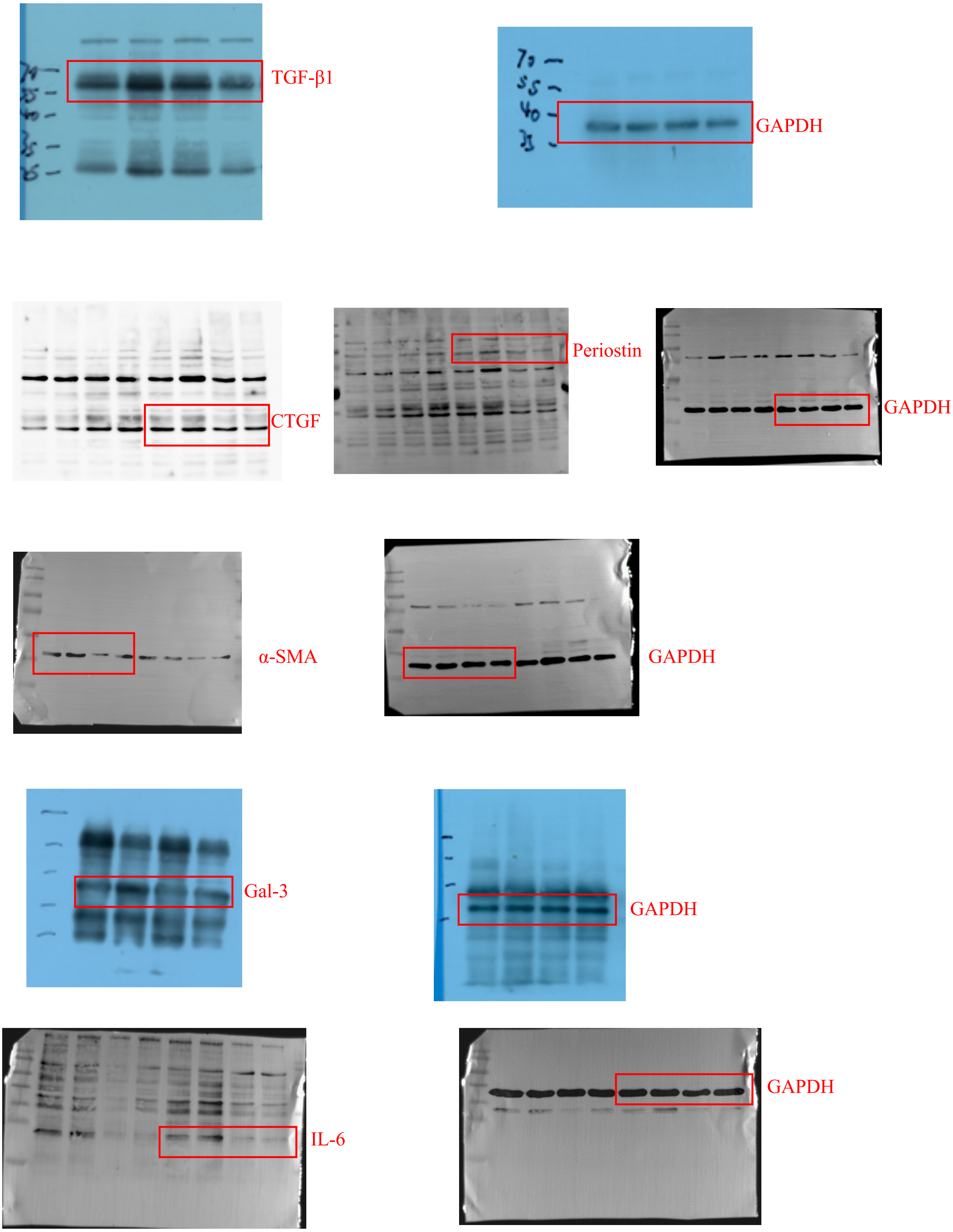
TGF-β1
GAPDH
CTGF
Periostin
GAPDH
α-SMA
GAPDH
Gal-3
GAPDH
IL-6
GAPDH

Figure 4H

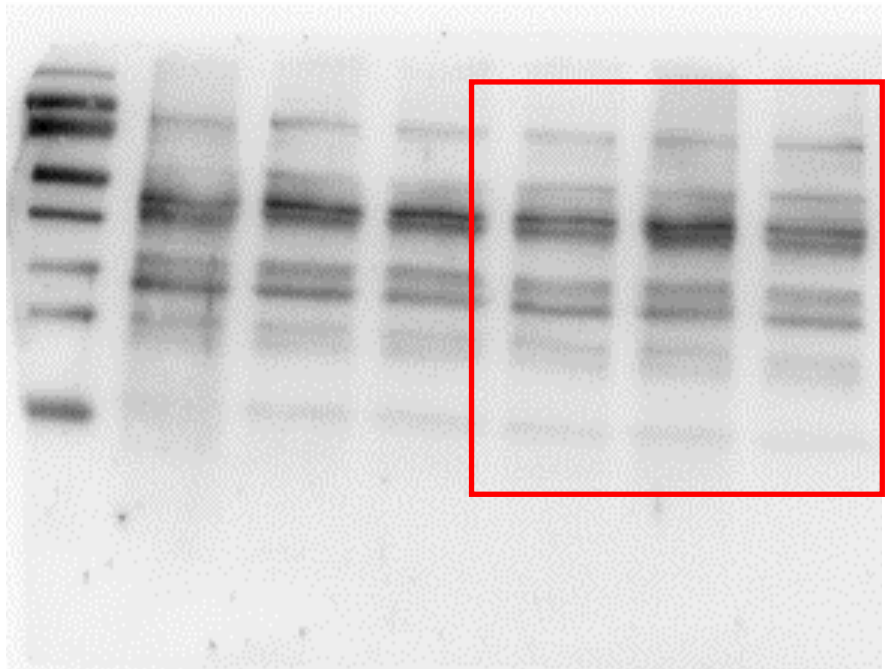

Pan-Kla

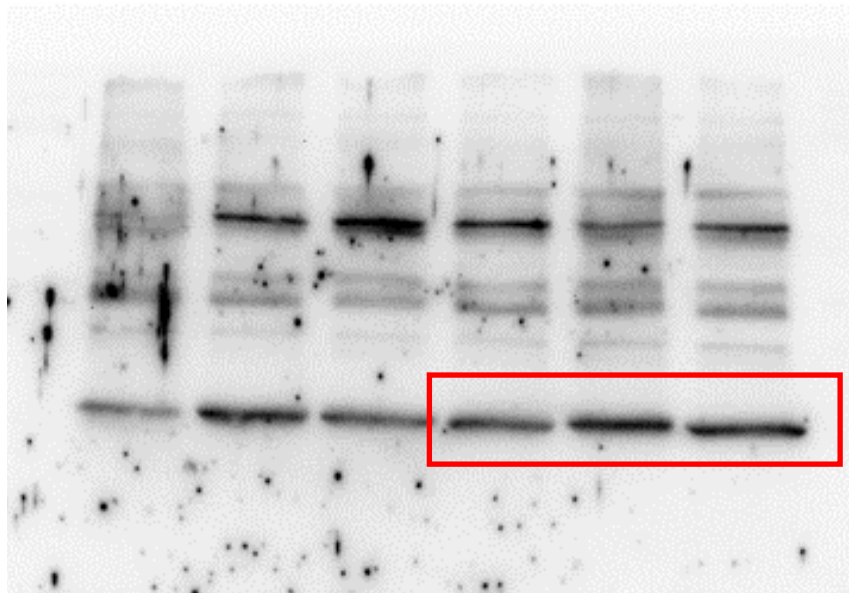

H3K18la

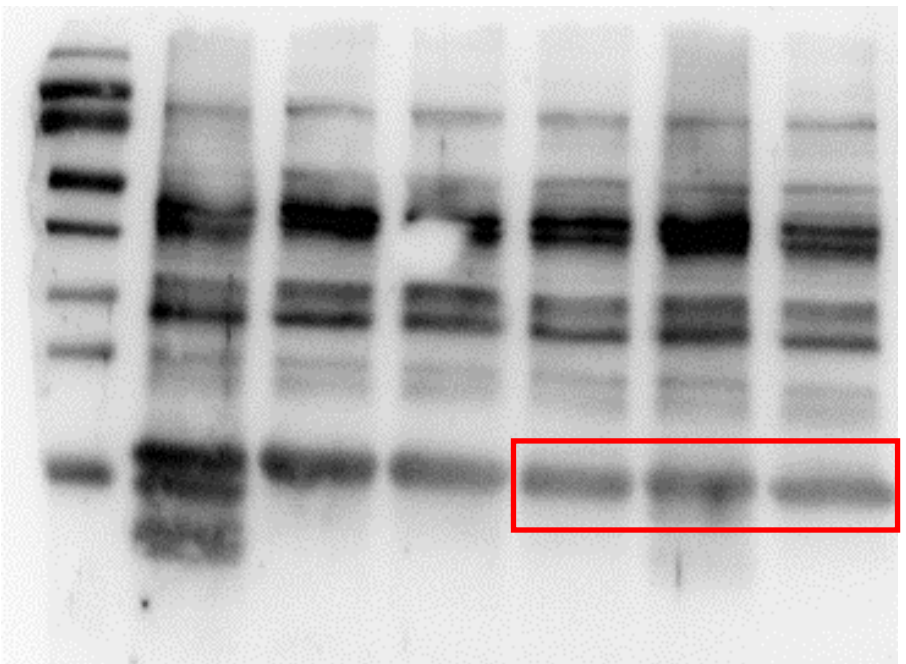

Histone H3

Figure 5E

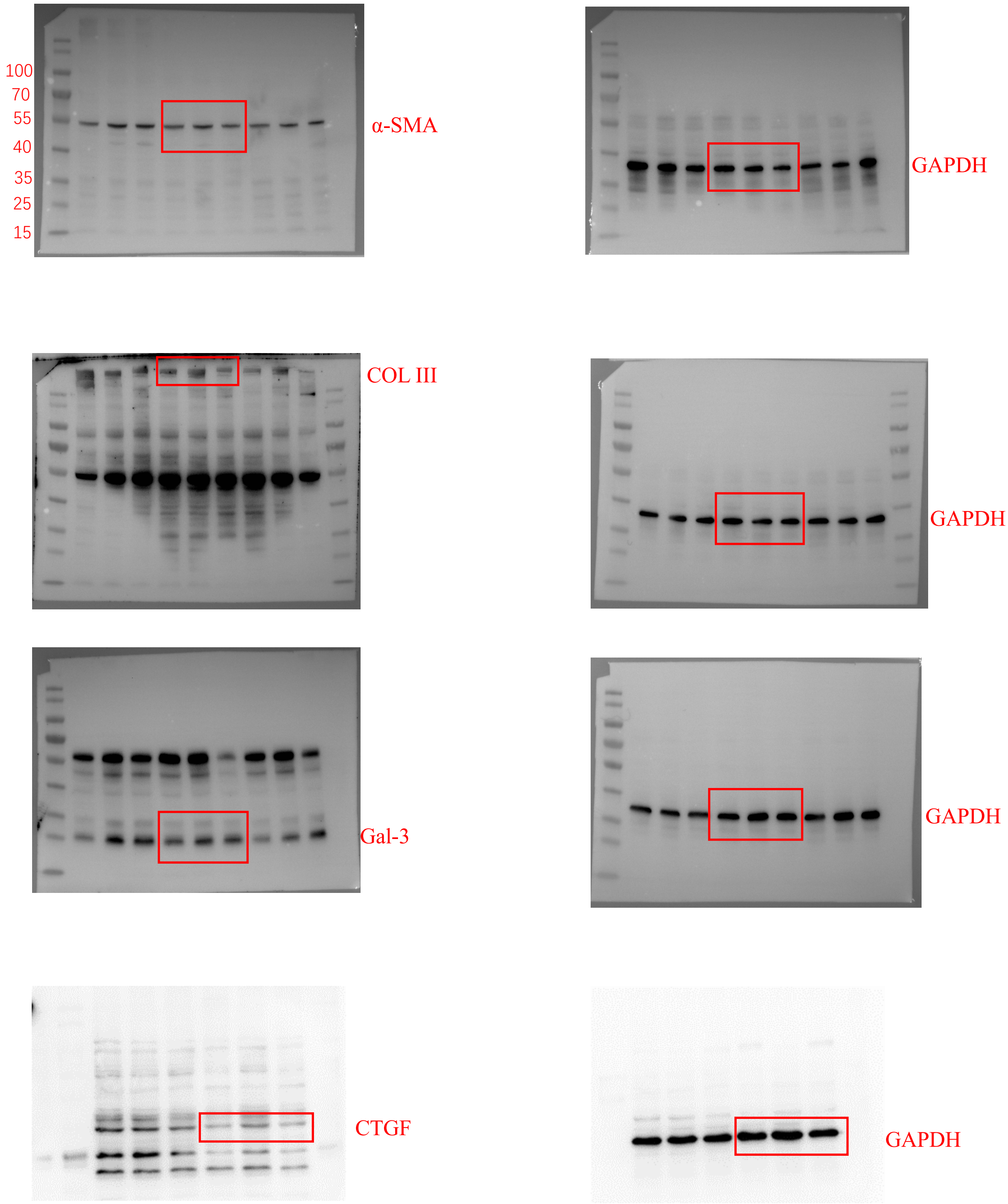
100
70
55
40
35
25
15
α-SMA
GAPDH
COL III
GAPDH
Gal-3
GAPDH
CTGF
GAPDH

Figure 5H

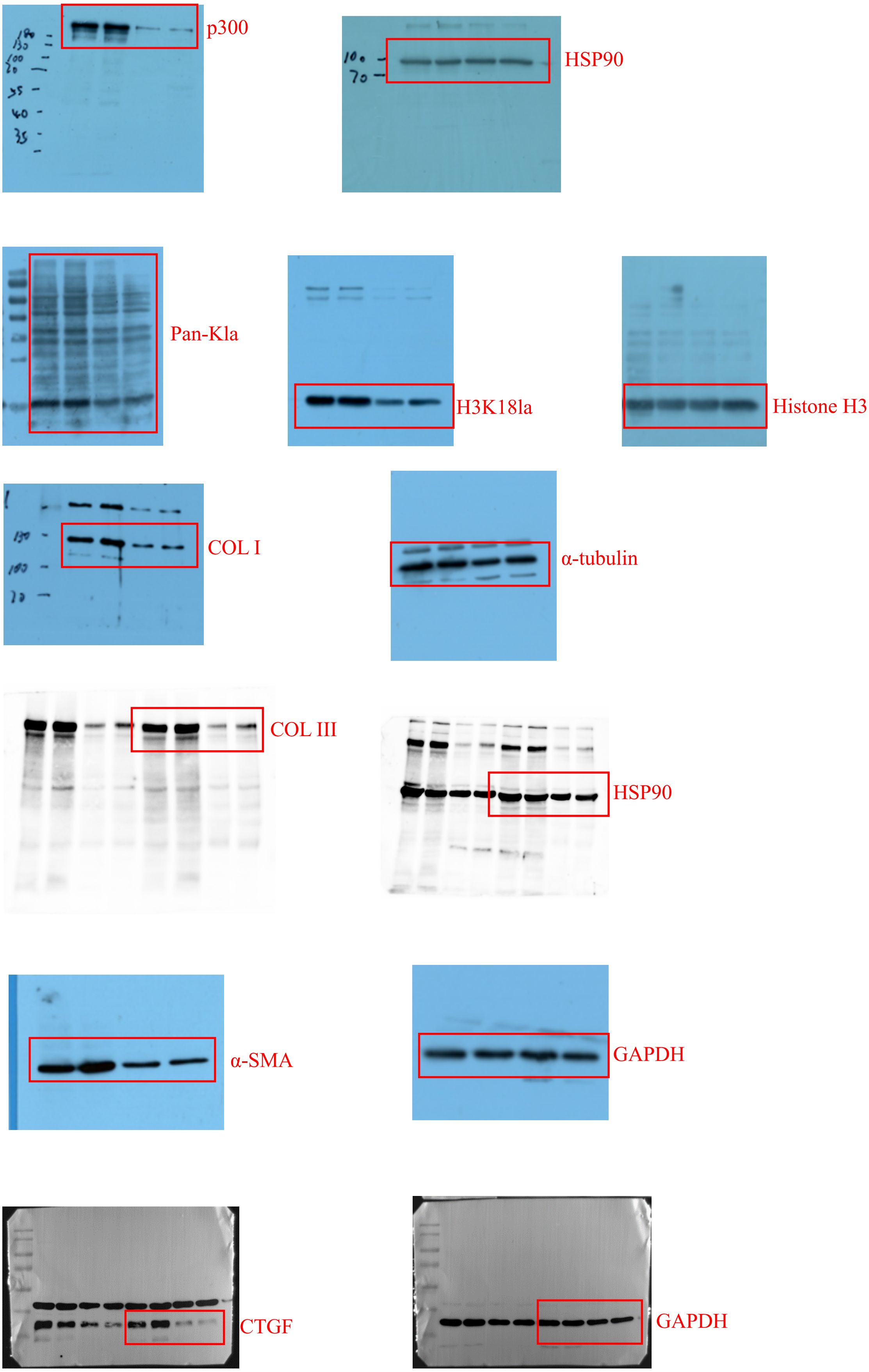
p300
HSP90
Pan-Kla
H3K18la
Histone H3
COL I
α-tubulin
COL III
HSP90
α-SMA
GAPDH
CTGF
GAPDH

Figure 6A-6C

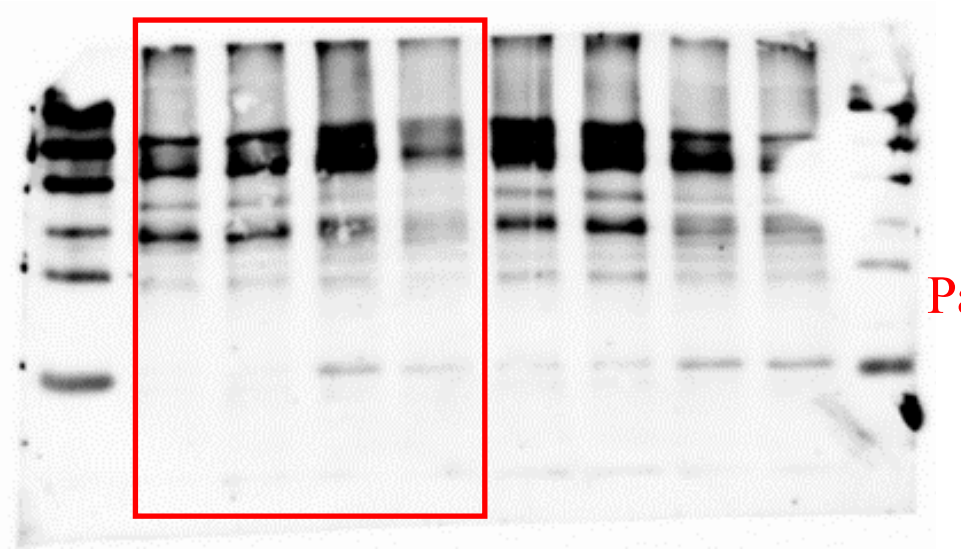

Pan-Kla

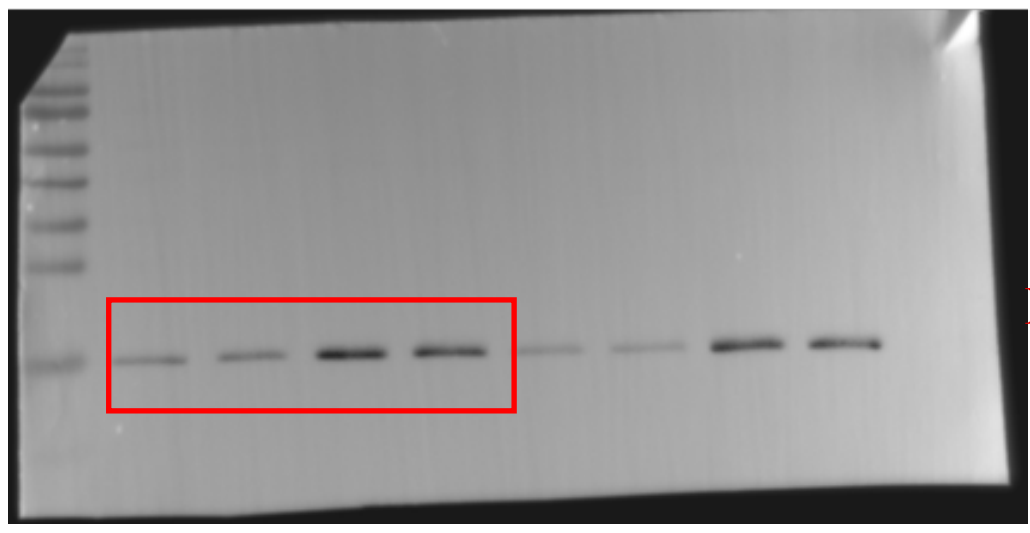

H3K18la

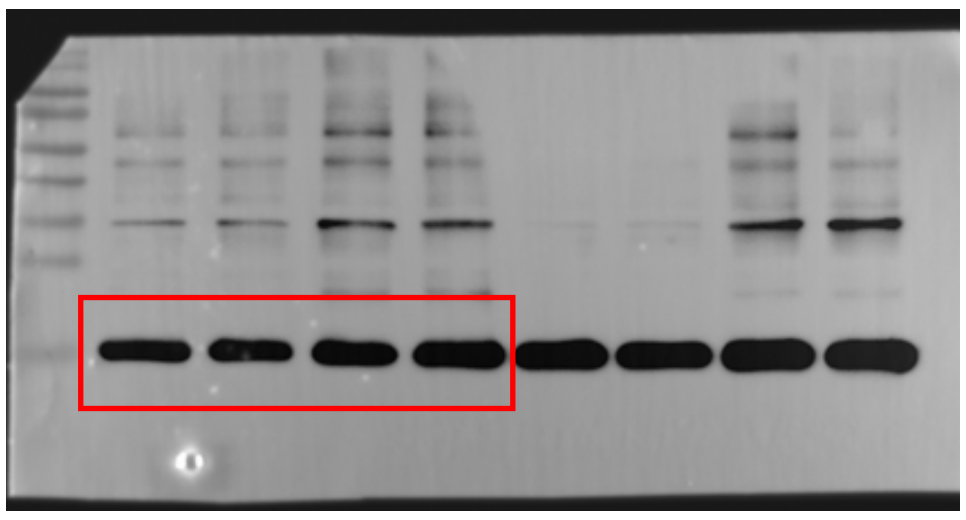

Histone H3

Figure 6F

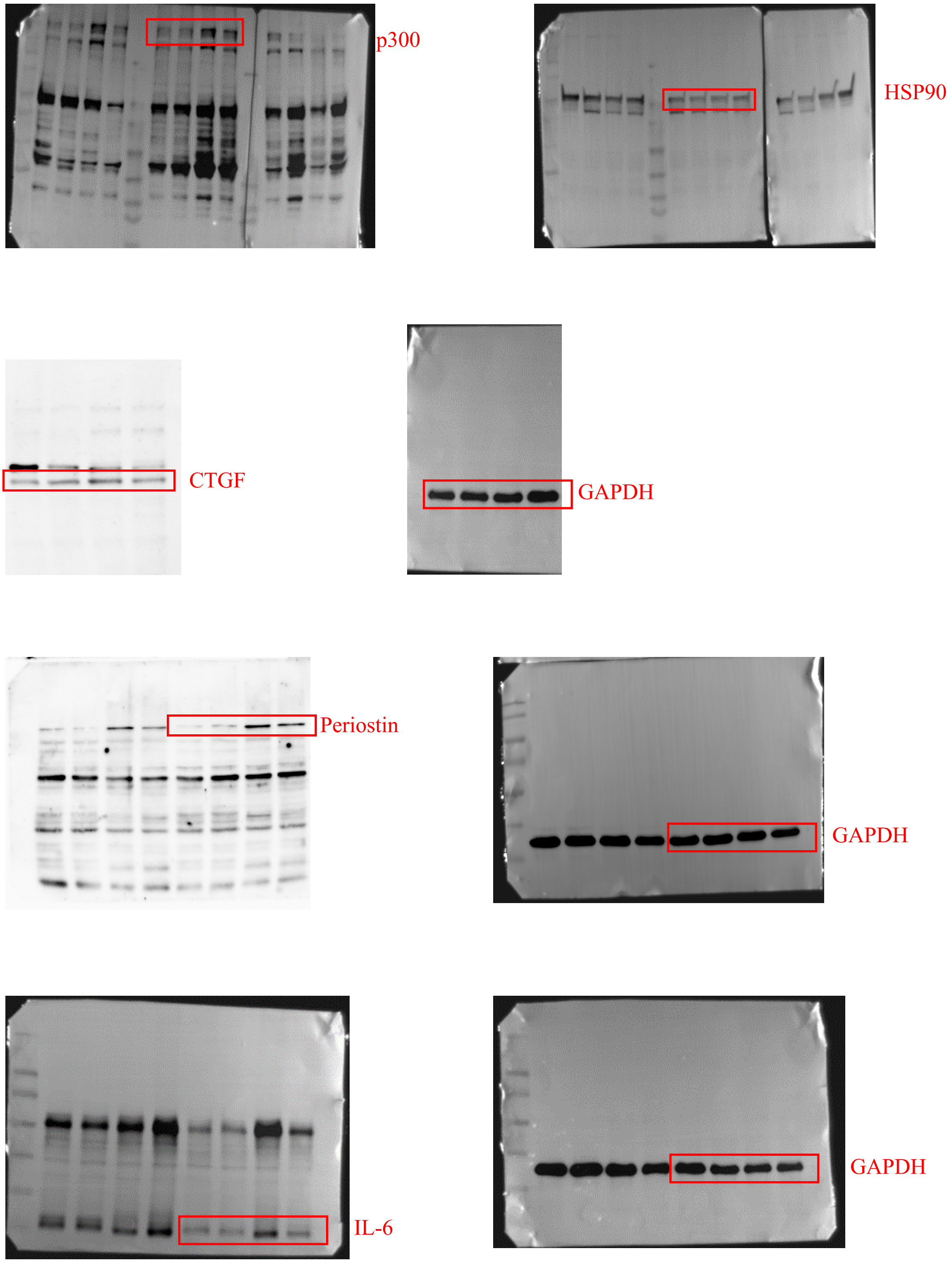

Figure 6I

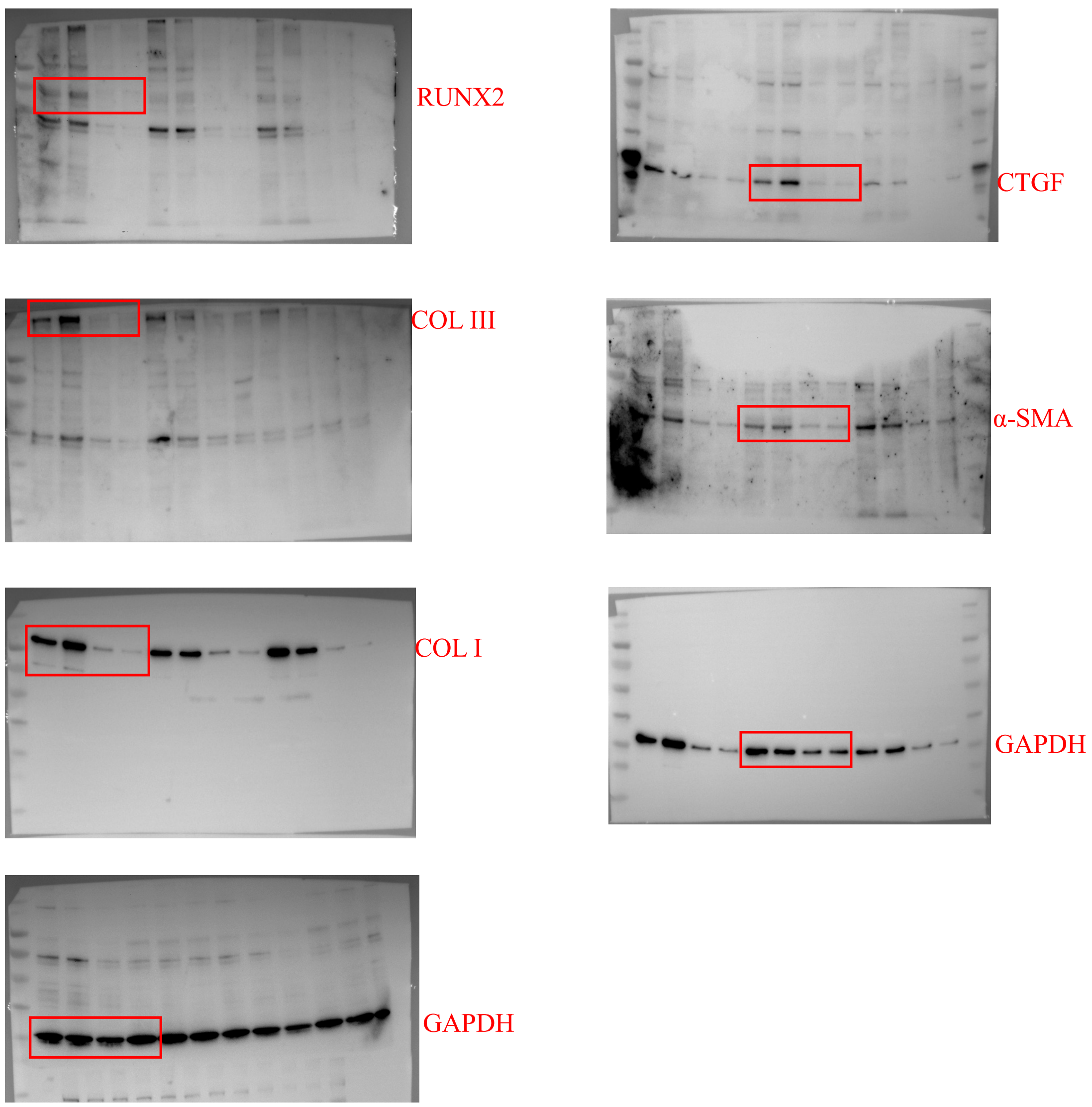

Figure 8F

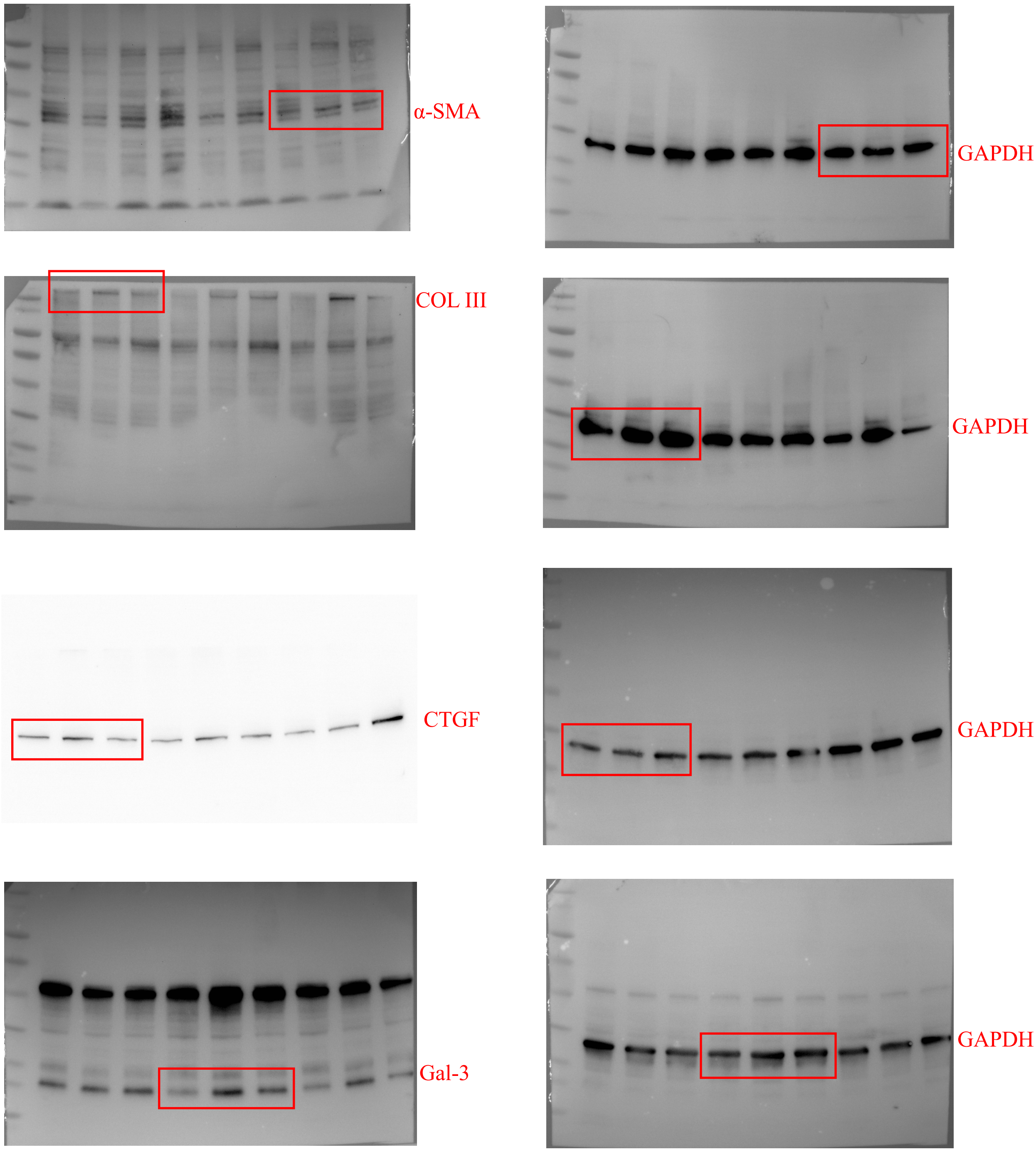

Figure 8J

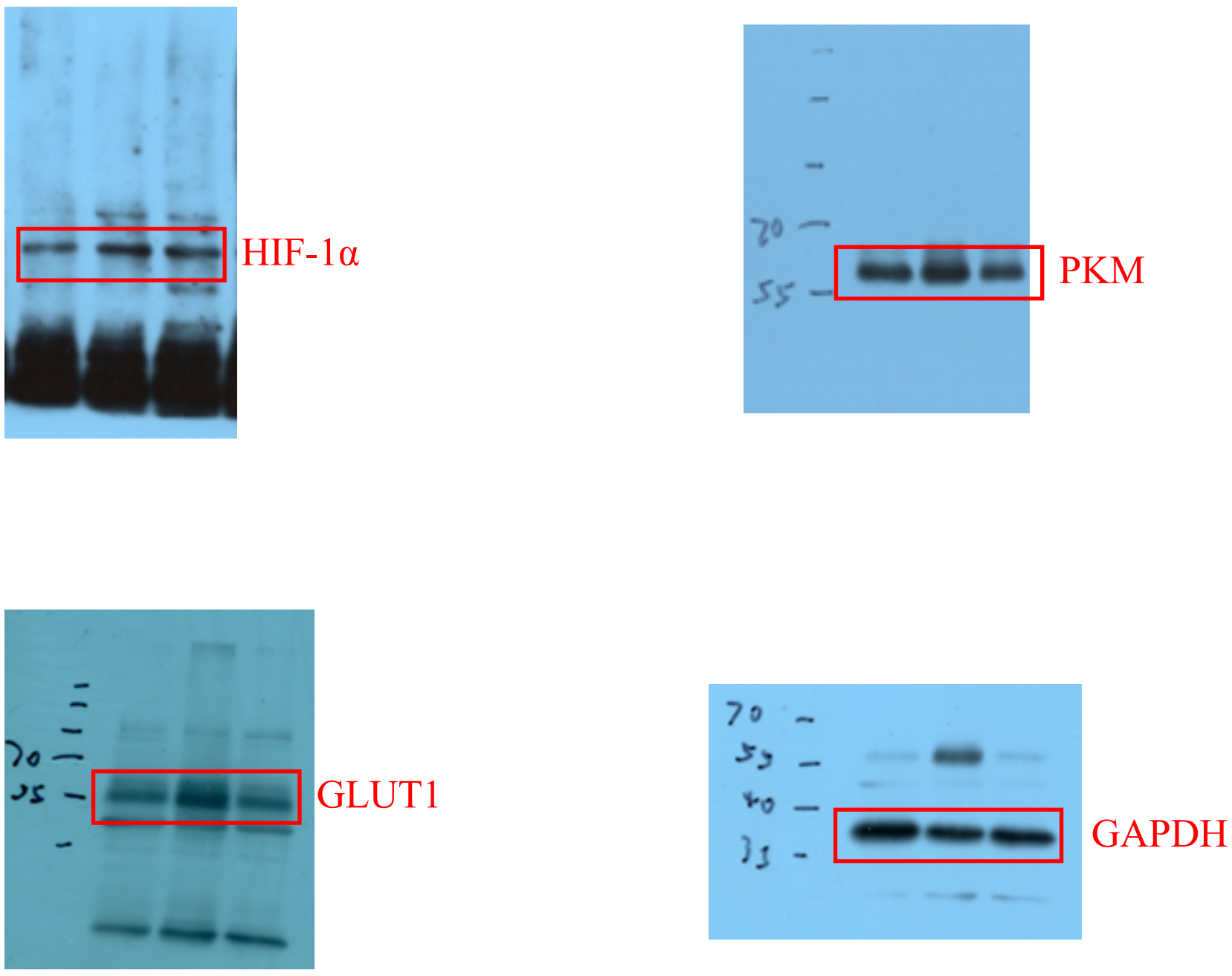

Figure S2B

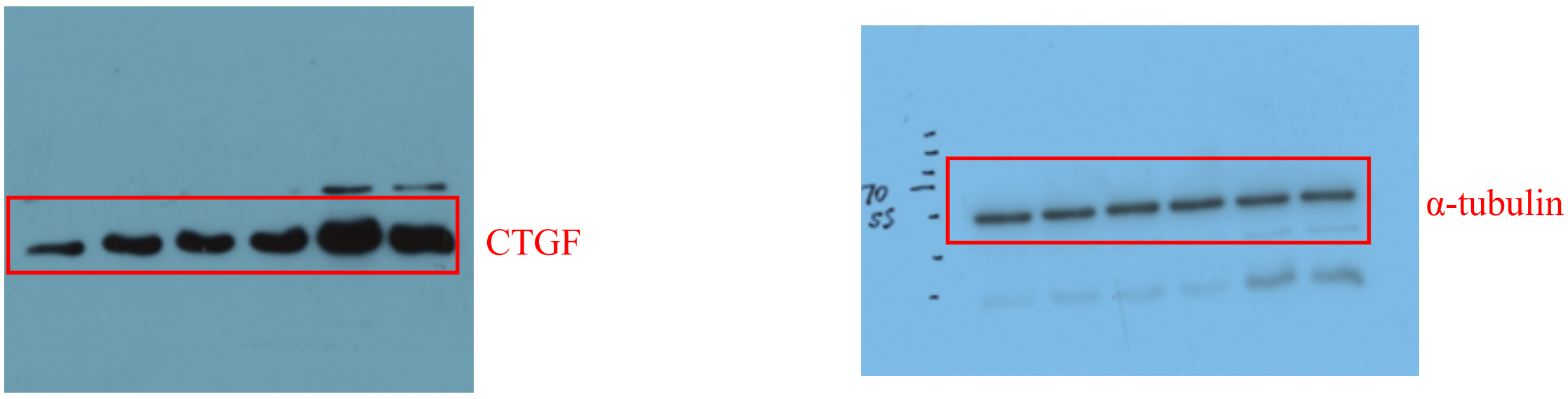

Figure S3B

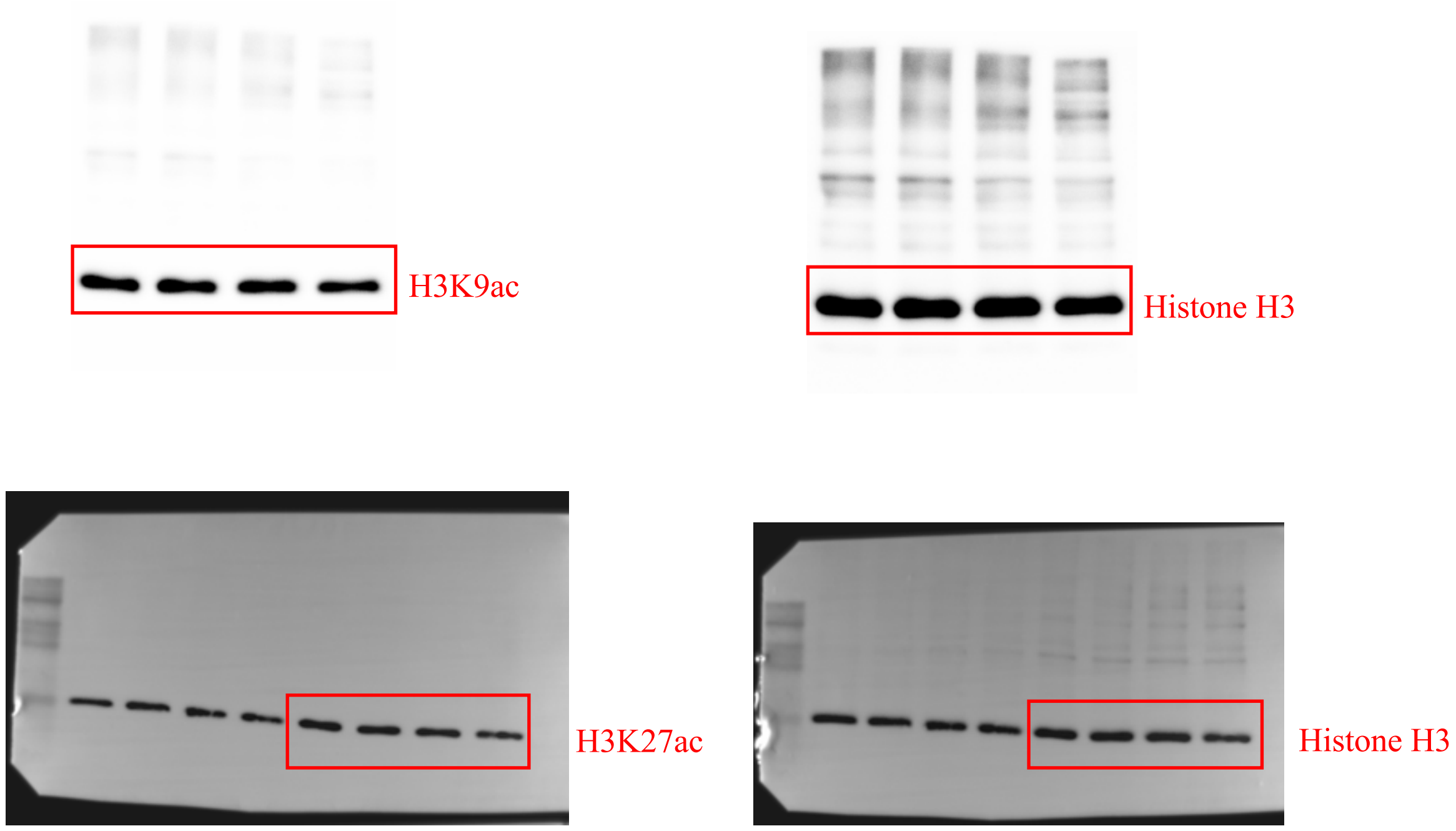

Figure S7
