# Supplementary material for: Inhibition of lactate-histone lactylation mediated cardiomyocyte-fibroblast communication mitigates cardiac fibrosis in cardiomyopathy models with Hippo pathway activation
Source: Cell Death Dis. 2026 Aug 1;17(1):922. doi: 10.1038/s41419-026-09137-7 (PMC13720063; doi:10.1038/s41419-026-09137-7)
Supplement: Supplementary file 2 [file 41419_2026_9137_MOESM1_ESM.docx]

**Inhibition of lactate-histone lactylation mediated cardiomyocyte-fibroblast communication mitigates cardiac fibrosis in cardiomyopathy models with Hippo pathway activation**

Li-Ye Jia^2#^; Xia-Xia Hai^1#^; Zhi-Qiang Wu^3^; Jing-Jing Li^1^; Yi-Yi Yang^1^; Meng-Chen Hou^4^; Lin-Hong Wu^1, 2^; Meng-Zhuan Han^1^; Yi Zhang^1^; Yungang Xu^3^; Jie Deng^5^; Bao-Chang Lai^7^; Junichi Sadoshima^8^; Xiu-Ling Deng^1^; Zheng-Da Pang^1*^; Xiao-Jun Du^1, 6*^; Gang She^1, 5*^

**Short title**: Inhibition of myocardial lactate metabolism mitigates cardiac fibrosis

^1^Department of Physiology and Pathophysiology, School of Basic Medical Sciences, Xi’an Jiaotong University Health Science Center, 76 West Yanta Road, Xi’an, 710061, Shaanxi, China

^2^School of Nursing and Rehabilitation, Xi’an Medical University, 1 Xinwang Road, Xi’an, 710021, Shaanxi, China

^3^Department of Cell Biology and Genetics, School of Basic Medical Sciences, Xi’an Jiaotong University Health Science Center, 76 West Yanta Road, Xi’an, 710061, Shaanxi, China

^4^Department of Pathology, Xi’an Guangren Hospital, Affiliated to Xi’an Jiaotong University Health Science Center, 21 Jiefang Road, Xi’an, 710005, Shaanxi, China

^5^Department of Cardiology, The Second Affiliated Hospital of Xi’an Jiaotong University, Xi’an, 710114, Shaanxi, China.

^6^Baker Heart and Diabetes Institute, 75 Commercial Road, Melbourne, Victoria 3004, Australia

^7^Cardiovascular Research Centre, School of Basic Medical Sciences, Xi’an Jiaotong University Health Science Center, 76 West Yanta Road, Xi’an, 710061, Shaanxi, China

^8^Department of Cell Biology and Molecular Medicine, Rutgers New Jersey Medical School, New Jersey, United States of America

^#^The 2 authors contributed equally to this work.

^*^Correspondence

1. Gang She, Email: shegang@xjtu.edu.cn

2. Xiao-Jun Du, Email: [xiao-jun.du@baker.edu.au](mailto:xiao-jun.du@baker.edu.au)

3. Zheng-Da Pang, Email: pangzd@mail.xjtu.edu.cn

**This file includes:**

Supplementary Table S1-S4

Supplementary Figure S1-S7

**Supplementary Table S1. Source and characteristics of antibodies used in this study.**

| **Target antigen** | **Source** | **Catalog** | **Working concentration** |
| --- | --- | --- | --- |
| Pan Kac | PTMbio | PTM-105RM | 1:1000 (WB) |
| H3K18ac | PTMbio | PTM-114RM | 1:1000 (WB) |
| H3K27ac | PTMbio | PTM-116RM | 1:1000 (WB) |
| H3K9ac | PTMbio | PTM-112RM | 1:1000 (WB) |
| Pan Kla | PTMbio | PTM-1401RM | 1:1000 (WB) |
| H3K18la | PTMbio | PTM-1427RM | 1:1000 (WB); 1:100 (CUT&Tag); 1:100 (IF) |
| H3K27la | PTMbio | PTM-1428 | 1:1000 (WB) |
| Histone H3 | Proteintech | 17168-1-AP | 1:2000 (WB) |
| PKM2 | Cell Signaling Technology | #4053 | 1:2000 (WB) |
| HK2 | Cell Signaling Technology | #55205 | 1:1000 (WB) |
| GLUT1 | Proteintech | 21829-1-AP | 1:2000 (WB) |
| HIF-1α | Cell Signaling Technology | #36169 | 1:1000 (WB) |
| MCT1 | Proteintech | 20139-1-AP | 1:1000 (WB) |
| MCT4 | Proteintech | 22787-1-AP | 1:1000 (WB) |
| LDHA | Proteintech | 19987-1-AP | 1:2000 (WB) |
| LDHB | Proteintech | 14824-1-AP | 1:1000 (WB) |
| TGF-β1 | Santa Cruz Biotechnology | sc-130348 | 1:500 (WB) |
| CTGF | Proteintech | 25474-1-AP | 1:1000 (WB) |
| Periostin | Abcam | ab152099 | 1:1000 (WB) |
| α-SMA | Santa Cruz Biotechnology | sc-32251 | 1:500 (WB); 1:100 (IF) |
| Galectin-3 | Santa Cruz Biotechnology | sc-32790 | 1:500 (WB) |
| IL-6 | Proteintech | 21865-1-AP | 1:2000 (WB) |
| COL I | Bioss | bs-10423R | 1:500 (WB) |
| COL III | Bioss | bs-0549R | 1:500 (WB) |
| p300 | Cell Signaling Technology | #86377 | 1:1000 (WB); 1:100 (IF) |
| RUNX2 | Cell Signaling Technology | #12556 | 1:1000 (WB); 1:100 (IF); 1:100 (CUT&RUN) |
| DDR2 | Santa Cruz Biotechnology | sc-81707 | 1:50 (IF) |
| HSP90 | Proteintech | 13171-1-AP | 1:2000 (WB) |
| α-Tubulin | Proteintech | 11224-1-AP | 1:2000 (WB) |
| GAPDH | Proteintech | HRP-60004 | 1:3000 (WB) |

**Supplementary Table S2.** **Specific primers in real-time PCR**

| **Name** | **Sequence** |
| --- | --- |
| *Fn1* | Forward: AGTGGCTGAAGTCGCAAGGAAAC |
|  | Reverse: TAAGTCTGGGTCACGGCTGTCTC |
| *Acta2* | Forward: TTCCTTCGTGACTACTGCCG |
|  | Reverse: TATAGGTGGTTTCGTGGATGCC |
| *Col1a1* | Forward: TTCTCCTGGCAAAGACGGAC |
|  | Reverse: CTCAAGGTCACGGTCACGAA |
| *Col3a1* | Forward: CATGCATAAATGCCAGCCCC |
|  | Reverse: AGGAGGGCCATAGCTGAACT |
| *Lgals3* | Forward: TAATCAGGTGAGCGGCACAG |
|  | Reverse: GGTAGGCCCCAGGATAAGCA |
| *Postn* | Forward: AAGGCGAAACGGTGACAGAA |
|  | Reverse: CCTCCTGTGGAAATCCTGGT |
| *Ccn2* | Forward: GACCCAACTATGATGCGAGCC |
|  | Reverse: ACTTAGCCCTGTATGTCTTCACA |
| *α-Tubulin* | Forward:TGGGCTCGCCTAGATCACAA |
|  | Reverse:GAAGCAGCACCTTGTGACATTT |

**Supplementary Table S3. Specific primers in RUNX2 CUT&RUN-qPCR**

| **Name** | **Sequence** |
| --- | --- |
| *Fn1* | Forward: GCCTATTGGCCCAGAGTCCG |
|  | Reverse: GCGGGAGATCAGCATCTCTTT |
| *Postn* | Forward: GATTGCTCAGAACCCAGGAGAT |
|  | Reverse: CTTGCGATGGCCTTACAGAA |
| *Acta2* | Forward: GGGACCTCAGCACAAAACTC |
|  | Reverse: CGGGAGCAGAACAGAGGAAT |
| *Tgfb1* | Forward: AGGTCCCCACTTCTAATACAGGA |
|  | Reverse: GGATTTGGGAGTTGTTGAAGGG |
| *Ccn2* | Forward: GTAGCCAGAACTGGCAAAGAGA |
|  | Reverse: GTTTCCAGCACAACACCAAGA |
| *Col1a1* | Forward: AGCACCTCTGGCCCATGTA |
|  | Reverse: AAGGGAAAGGAGTCCACCCA |

**Supplementary Table S4. Organ weight and heart function in male and female nTG and Mst1-cTG mice at 4.5 months of age.**

|  | Non-TG | | Mst1-cTG | |
| --- | --- | --- | --- | --- |
| **Organ weight** | male (n=15) | female (n=10) | male (n=14) | female (n=15) |
| Body weight (g) | 40.2±0.9~ | 25.8±0.5 | 41.5±0.9~ | 26.1±0.4 |
| Tibia length (TL, mm) | 17.3±0.1 | 17.2±0.1 | 17.4±0.1 | 17.3±0.1 |
| Heart weight (HW, mg) | 156.1±2.9~ | 114.0±2.7 | 166.1±3.4~* | 137.9±3.7* |
| HW/TL (mg/mm) | 9.02±0.1~ | 6.61±0.17 | 9.56±0.17~* | 7.94±0.17* |
| Atrial weight (AW, mg) | 9.3±0.5~ | 6.4±0.2 | 15.3±0.7* | 13.7±0.7* |
| AW/TL (mg/mm) | 0.54±0.03~ | 0.37±0.02 | 0.88±0.04* | 0.79±0.04* |
| Lung weight (LW, mg) | 155.6±3.0 | 149.6±1.9 | 180.1±4.3* | 179.3±5.3* |
| LW/TL (mg/mm) | 8.99±0.17 | 8.68±0.11 | 10.38±0.26* | 10.34±0.29* |
| **Heart function** | male (n=9) | female (n=11) | male (n=8) | female (n=9) |
| Heart rate (beats/min) | 551±11 | 506±17 | 450±14* | 476±14 |
| LVESD (mm) | 1.85±0.04 | 2.07±0.04 | 2.98±0.12* | 3.00±0.05* |
| LVEDD (mm) | 3.79±0.05 | 3.93±0.06 | 4.62±0.10* | 4.53±0.05* |
| LV-PW (mm) | 0.98±0.07~ | 0.83±0.06 | 0.70±0.03* | 0.77±0.02 |
| IVS (mm) | 0.98±0.08~ | 0.80±0.03 | 0.73±0.02 * | 0.78±0.02 |
| Fractional shortening (%) | 51±1 | 47±1 | 36±2*^ | 34±1*^ |

~ *P*<0.05 compared with female of same genotype. * *P*<0.05 compared with nTG of same gender.

LVESD and LVEDD: Left ventricular end-systolic or end-diastolic dimension. LV-PW: LV posterior wall; IVS: intra-ventricular septum.

**Supplementary Figures**

**Figure S1. Marker genes and cell type clustering of SnRNA-seq in Mst1-TG mouse heart. *A.*** UMAP projection of all mouse cardiac cell clusters. ***B.*** The marker genes defining 13 main cell types of cell cluster. C. The marker genes defining ventricular or atrial cardiomyocyte (left) and the marker genes of oxidative stress and dilated cardiomyopathy used for sub-clustering cardiomyocytes by stress degree (right).

**Figure S2. Effect of HIF-1α inhibitor PX-478 treatment on the expression of glycolytic gene and protein in Mst1-TG mouse heart. *A:*** Expression of glycolytic genes in LV tissues by RT-qPCR. ***B:*** Western blotting images and mean values of glycolytic protein level in the LVs. Data are presented as Mean ± SEM, n=5-8, ^**^*P*<0.01, ^***^*P*<0.001 *vs.* nTG; ^#^*P*<0.05, ^##^*P*<0.01 *vs.* Mst1-TG.

**Figure S3. Effect of different concentration of lactate on cardiac fibroblast viability and CTGF expression. *A.*** Cell viability tested by MTT at different concentration of lactate stimulation in cultured cardiac fibroblast (n=12). ***B.*** Western blotting images and mean values of CTGF protein level in the fibroblasts treated by different concentration of lactate (n=5). Data are presented as Mean ± SEM, ^*^*P*<0.05, ^**^*P*<0.01, ^***^*P*<0.001 *vs.* CTL.

**Figure S4. Validation the efficiency of fibroblast p300 gene knock-down mediated by AAV9 virus in Mst1-TG mouse myocardium.** Representative immunofluorescent staining of p300 (red) and DDR2 (green) in mouse LV sections, cell nuclei were stained with DAPI (blue).

**Figure S5. Effects of fibroblast p300 gene knock-down on organ weight in Mst1-TG mouse heart.** The ratio of mouse heart weight (HW) to tibia length (TL) and the ratio of mouse organ weight (heart, lung, liver, kidney and spleen) to body weight. Data are presented as Mean ± SEM, n=7-8, ^*^*P*<0.05, ^***^*P*<0.001 *vs.* nTG+AAV9-CTL shRNA; ^#^*P*<0.05, ^##^*P*<0.01, ^###^*P*<0.001 *vs.* Mst1-TG+AAV9-CTL shRNA.

**Figure S6. Effects of fibroblast p300 gene knock-down on fibroblast H3K18 level in Mst1-TG mouse myocardium.** Representative immunofluorescent staining of H3K18la (red) and DDR2 (green) in mouse LV sections, cell nuclei were stained with DAPI (blue).

**Figure S7. Effects of fibroblast p300 gene knock-down on** **H3K9 and H3K27 acetylation level in Mst1-TG mouse heart.** Western blotting images and mean values of H3K9ac and H3K27ac level in the mouse LVs (n=8). Data are presented as Mean ± SEM.
